# Liraglutide in Acute Minor Ischemic Stroke or High-Risk Transient Ischemic Attack With Type 2 Diabetes: The LAMP Randomized Clinical Trial

**DOI:** 10.1001/jamainternmed.2025.5684

**Published:** 2025-11-03

**Authors:** Huili Zhu, Bin Yang, Longyan Lu, Yufeng Li, Rubo Sui, Kewei Liu, Suling Tan, Lihua Wang, Jianmin Qiu, Jianbin Zhong, Tongguo Wei, Yuzhang Bei, Jianmin Huang, Suping Zhang, Yan Ji, Wenjun Wu, Youjia Li, Ying Huang, Yangkun Chen, Xiaoyun Huang, Guoyong Zeng, Yusheng Zhang, Lian Huang, Hao Li, Xiangbing Wang, Yongjun Wang, Anding Xu

**Affiliations:** 1 Department of Neurology, the First Affiliated Hospital of Jinan University, Guangzhou, China; 2 Department of Neurology, the First Affiliated Hospital of Jinzhou Medical University, Jinzhou, China; 3 Department of Neurology, People’s Hospital of Longmen County, Longmen, China; 4 Department of Neurology, People’s Hospital of Xinfeng County, Xinfeng, China; 5 Department of Neurology, the Second Affiliated Hospital of Harbin Medical University, Harbin. China; 6 Department of Neurology, the First Hospital of Putian, Putian, China; 7 Department of Neurology, the Fourth Affiliated Hospital of Guangzhou Medical University, Guangzhou, China; 8 Department of Neurology, Meizhou People’s Hospital, Meizhou, China; 9 Department of Neurology, Liuyang Jili Hospital, Liuyang, China; 10 Department of Neurology, Affiliated Hospital of Youjiang Medical College for Nationalities, Baise, China; 11 Department of Neurology, Guangzhou Red Cross Hospital, Guangzhou, China; 12 Department of Neurology, Guangdong Clifford Hospital, Guangzhou, China; 13 Department of Neurology, Zhongshan People’s Hospital, Zhongshan, China; 14 Department of Neurology, the First People’s Hospital of Zhaoqing, Zhaoqing, China; 15 Department of Neurology, the First Affiliated Hospital of Gannan Medical College, Ganzhou, China; 16 Department of Neurology, Dongguan People’s Hospital, Dongguan, China; 17 Department of Neurology, Houjie Hospital, Dongguan City, Dongguan, China; 18 Department of Neurology, Ganzhou people’s Hospital, Ganzhou, China; 19 Beijing Tiantan Hospital, Capital Medical University, Beijing, China; 20 Rutgers Robert Wood Johnson Medical School, New Brunswick, New Jersy

## Abstract

**Importance:**

Glucagon-like peptide–1 receptor agonists can reduce the risk of cardiovascular events in patients with high-risk type 2 diabetes (T2D). However, dedicated randomized clinical trials that evaluate their efficacy in acute ischemic stroke are lacking.

**Objective:**

To investigate the safety and efficacy of liraglutide in patients with T2D and minor acute ischemic stroke (AIS) or high-risk transient ischemic attack (TIA).

**Design, Setting, and Participants:**

The LAMP trial was designed as a multicenter, controlled, prospective, randomized, open-label, blinded end point trial and conducted at 27 hospitals in China from June 25, 2019, through December 27, 2023. The final follow-up was on March 24, 2024. Data were analyzed on May 1, 2024. The study included patients with T2D who had minor AIS (National Institutes of Health Stroke Scale score ≤3) or high-risk TIA (ABCD^2^ score [age, blood pressure elevation on first assessment after TIA, unilateral weakness, speech disturbance, duration of symptoms and diabetes] ≥4).

**Interventions:**

Eligible patients were randomized within 24 hours of symptom onset to the liraglutide group and the control group. Both groups received standard treatment according to the guidelines. The liraglutide group received liraglutide once daily for 90 days (0.6 mg for the first week, which was increased to 1.2 mg in the second week and followed by 1.8 mg until day 90) in addition to standard therapy.

**Main Outcomes and Measures:**

The primary outcome was stroke recurrence (ischemic or hemorrhagic) after 90 days, and the safety outcome was symptomatic intracranial hemorrhage and all-cause mortality at 90 days.

**Results:**

In all, 636 patients (median [IQR] age, 63.5 [57.8-70.0] years; 231 female individuals [36.3%]) were randomized. Within 90 days, 25 patients (7.9%) in the liraglutide group and 44 (13.8%) in the control group experienced stroke recurrence (hazard ratio, 0.56; 95% CI, 0.34-0.91; *P* = .02). A significantly higher proportion of patients (modified Rankin Scale score, ≤1) in the liraglutide group (274 [87.3%]) than in the control group (246 [77.8%]) achieved excellent functional outcomes (odds ratio, 1.95; 95% CI, 1.28-3.00; *P* = .002). The rates of symptomatic intracranial hemorrhage and all-cause mortality were low and similar between the groups.

**Conclusions and Relevance:**

The trial results suggest that among Chinese patients with T2D and minor AIS or high-risk TIA, liraglutide treatment might reduce stroke recurrence and improve 90-day outcomes. However, given the underpowered nature of the study, these findings should be interpreted with caution.

**Trial Registration:**

ClinicalTrials.gov Identifier: NCT03948347

## Key Points

**Question:**

Do glucagon-like peptide–1 receptor agonists (liraglutide) reduce stroke recurrence in patients with type 2 diabetes who have minor acute ischemic stroke or high-risk transient ischemic attack?

**Findings:**

In this randomized clinical trial of 636 participants with type 2 diabetes, 25 patients (7.9%) who were randomly assigned to receive standard therapy plus liraglutide and 44 (13.8%) of those who received standard therapy had recurrent stroke at 90 days.

**Meaning:**

The results of this randomized clinical trial suggest that, compared with standard therapy, liraglutide might reduce recurrent stroke in patients with type 2 diabetes who had minor acute ischemic stroke or high-risk transient ischemic attack.

## Teaser

This randomized clinical trial examines the safety and efficacy of liraglutide in patients with type 2 diabetes and minor acute ischemic stroke or high-risk transient ischemic attack.

## Introduction

Minor acute ischemic stroke (AIS) or high-risk transient ischemic attack (TIA) is associated with a high stroke recurrence rate. Despite adherence to guideline-based therapies, residual risk of recurrence persists, with a 5% to 8% stroke recurrence within 90 days.^1,2^ Type 2 diabetes (T2D) is a primary risk factor for ischemic cerebrovascular disease.^3^ Approximately 33% of the individuals who have experienced a stroke also have diabetes, resulting in an elevated residual risk of stroke recurrence.^2,4^ Among patients with minor AIS who had T2D, the 90-day recurrence rate is 12.8%.^5^ However, intensive glycemic control using insulin or sulfonylureas does not substantially reduce the risk of macrovascular complications.^6,7^ These findings suggest that, in addition to hyperglycemia, other pathogenic mechanisms, such as insulin resistance, inflammatory responses, and oxidative stress, may contribute to ischemic stroke recurrence.

Glucagon-like peptide–1 (GLP-1), an incretin hormone, stimulates insulin release in a glucose-dependent manner and inhibits glucagon secretion from the pancreas.^8^ While GLP-1 receptor agonists (GLP-1RAs) reduce the incidence of major adverse cardiovascular events in patients with T2D at high cardiovascular risk, there is still insufficient evidence regarding their efficacy in preventing stroke recurrence and improving outcomes during the acute phase of ischemic stroke.^9,10,11,12^ In addition to managing hyperglycemia, GLP-1RAs also improve insulin resistance, reduce inflammatory responses, and slow the progression of atherosclerosis.^13,14^ Liraglutide, a widely used once-daily GLP-1RA for managing T2D, promotes weight loss and provides protection against macrovascular and microvascular complications in patients with diabetes.^10,15^ Liraglutide has improved neurological outcomes in rats with acute focal cerebral ischemia.^16^ These findings indicate that GLP-1RAs may reduce the residual risk of recurrence and clinical prognosis of patients with AIS. However, clinical trials on the use of GLP-1RAs in these patients are lacking. In the Liraglutide in Acute Minor Ischemic Stroke or High-Risk Transient Ischemic Attack Patients With Type 2 Diabetes (LAMP) trial, we assessed the safety and effectiveness of liraglutide in reducing stroke recurrence and improving the prognosis of patients with minor AIS or high-risk TIA.

## Methods

### Study Design

The LAMP trial, a multicenter, controlled, prospective, randomized, open-label, blinded end point trial, assessed the safety and effectiveness of liraglutide in reducing stroke recurrence and improving the prognosis of patients with minor AIS or high-risk TIA with T2D (see Supplement 1 for the trial protocol and Supplement 2 for the statistical analysis plan). The trial, conducted between June 25, 2019, and December 27, 2023, at 27 sites (eAppendix 1 in Supplement 3) in China, was approved by the ethics committee of the First Affiliated Hospital of Jinan University and other participating hospitals. Written informed consent was obtained from all patients or their legally authorized representatives. This study followed the Consolidated Standards of Reporting Trials (CONSORT) reporting guidelines for randomized clinical trials.

An independent data monitoring committee that was composed of 2 experienced biostatisticians and 1 senior neurologist reviewed unmasked safety data and trial conduct every 6 months to ensure safety and data integrity. A separate, masked clinical event committee of 3 senior neurologists adjudicated all efficacy and safety end points. A steering committee oversaw trial operations, ensuring protocol adherence and managing strategic and administrative matters without access to treatment assignments or end point adjudication. Further details are available in eAppendix 2 of Supplement 3.

### Participants

Eligibility entailed that participants were 50 years or older, had minor AIS with a National Institutes of Health Stroke Scale score of 3 or less (range, 0-42; higher scores indicate more severe stroke) or high-risk TIA as determined by an ABCD^2^ score (age, blood pressure elevation on first assessment after TIA, unilateral weakness, speech disturbance, duration of symptoms and diabetes) of 4 or greater (range, 0-7; higher scores indicate a higher risk of stroke), were within 24 hours of symptom onset, and had either known or newly diagnosed T2D. Known T2D diagnosis was confirmed by medical records and/or ongoing antidiabetic therapy; diagnostic criteria for new cases are in eTable 1 in Supplement 3. For patients with stroke history, no sequelae should be present, indicated by a modified Rankin Scale (mRS) score of 0 to 1 (range, 0-6, with 0-1 denoting no disability, 2-5 representing increasing levels of disability, and 6 indicating death).

Exclusion criteria included diagnosis of intracranial hemorrhage on computed tomography, iatrogenic or cardiogenic stroke, thrombolysis or endovascular treatment, or treatment with GLP-1RAs within the previous 3 months. Full criteria are in the eMethods in Supplement 3.

### Randomization and Masking

After providing written informed consent, patients were randomized 1:1 to liraglutide or control using block randomization (block size 4) with sealed, opaque, numbered envelopes. Only those responsible for evaluating the results were unaware of the allocations. The statisticians who performed the final analysis were masked to the group allocation.

### Procedures

Liraglutide was initiated within 1 hour of randomization at 0.6 mg per day, which was increased to 1.2 mg in week 2 and 1.8 mg in week 3 via subcutaneous injection and then maintained at 1.8 mg per day until day 90. If dose escalation resulted in intolerable adverse effects, intervals for dose escalation were extended, and maintenance doses less than the target dose of 1.8 mg per day were considered. Dipeptidyl peptidase–IV inhibitors and sodium-glucose cotransporter-2 inhibitors were prohibited in both groups. The secondary prevention medications for AIS, as well as blood pressure and blood glucose management, were all administered per 2018 Guidelines for the Early Management of Patients With Acute Ischemic Stroke from the American Heart Association/American Stroke Association, the 2014 China Secondary Prevention Guidelines for Ischemic Stroke and Transient Ischemic Attack, and the 2017 China Diabetes Prevention Guidelines.^17,18,19^ Blood glucose control targets were set to a range of 140 to 180 mg/dL (to convert to mmol/L, multiply by 0.0555) in both arms and were monitored to prevent hypoglycemia throughout the study period. Demographic and clinical characteristics were obtained after randomization. Follow-up data were collected at a mean (SD) days of 7 (1), 30 (3), and 90 (7) days after randomization.

### Outcomes

Masked centralized follow-up was conducted by independently trained physicians. The primary outcome was 90-day stroke recurrence (ischemic or hemorrhagic) after 90 days. Ischemic stroke was defined as an event of neurological dysfunction resulting from a focal infarction in the brain, spinal cord, or retina. Any of the following criteria were indicative of a new ischemic stroke: (1) new focal neurological deficits lasting less than 24 hours that were not attributable to nonischemic causes, with neuroimaging evidence of a new cerebral infarction or (2) new focal neurological deficits lasting 24 hours or longer with or without imaging evidence that were not attributable to nonischemic causes.^1^ Hemorrhagic stroke was defined as rapidly progressing neurological dysfunction symptoms caused by the accumulation of blood in the nontraumatic brain parenchyma, ventricular system, or subarachnoid space (eTable 2 in Supplement 3). Secondary outcomes included the percentage of new clinical vascular events (eg, ischemic stroke, hemorrhagic stroke, TIA, myocardial infarction, and vascular death) and excellent functional outcomes (mRS ≤1) and favorable functional outcomes (mRS ≤2) at 90 days. Safety outcomes included symptomatic intracranial hemorrhage (sICH) as defined by the European Cooperative Acute Stroke Study II,^20^ hypoglycemia, gastrointestinal disorders, pneumonia, pancreatitis, and all-cause death within 90 days. Hypoglycemia was defined as a self-monitored blood glucose level less than 70 mg/dL, based on patient-recorded home glucose measurements that were recorded in logbooks or applications and reviewed at follow-ups.

### Sample Size Calculation

Based on the Clopidogrel With Aspirin in Acute Minor Stroke or Transient Ischemic Attack trial, the 90-day risk of stroke recurrence in the dual antiplatelet group was 12.8% among patients with T2D who had high-risk TIA or minor stroke and were treated within 24 hours of symptom onset.^5^ Previous randomized clinical trials demonstrated that GLP-1RAs reduce the risk of nonfatal stroke by 11% to 39% in patients with high-risk T2D.^9,10,11,12^ The sample size formula was based on a comparison of the proportions in both groups. We hypothesized that the risk of stroke recurrence would decrease by 33% in the liraglutide-treated group. Therefore, a sample size of 1708 patients would have 80% power to detect a 2-sided α error of .05, with 5% patient loss.

### Statistical Analyses

Efficacy and safety were analyzed in the intention-to-treat (ITT) population, including all randomized patients regardless of treatment adherence or protocol violations. The ITT and per-protocol analyses for primary and secondary outcomes used consistent methods. The safety population comprised all randomized patients who received at least 1 dose of the study drug and did not withdraw their consent and was used for adverse event analysis. Comprehensive definitions of all analyzed populations are presented in Supplement 2. Measurement data between the groups were compared using 2 independent sample *t* tests, and mean (SD) was used for statistical description. If data distribution was non-normal, we performed the Mann-Whitney *U* test. χ^2^ and Fisher exact tests were performed to compare categorical data between the groups, and percentages were used for statistical description. The Kaplan-Meier analysis was performed to estimate the cumulative stroke risk (ischemic or hemorrhagic) for the 90-day follow-up, with hazard ratios (HRs) and 95% CIs determined using Cox proportional hazards methods to evaluate the treatment effect, with trial centers included as a random effect. When multiple events of the same type occurred, the time to the first event was considered. Patients without a primary outcome were censored at death, last known contact, or at 90 days, whichever occurred first. Data before drug administration were used as baseline data. Similar approaches were used to compare the secondary outcomes of clinical vascular events and safety outcomes of sICH, hypoglycemic events, gastrointestinal disorders, pneumonia, acute pancreatitis, and death.

The proportions of mRS scores of 0 to 1 and mRS scores of 0 to 2 at 90 days between the groups were compared using a binary logistic analysis with calculated odds ratios (ORs) and 95% CIs. A 2-sided *P* < .05 was considered significant. The primary outcome was also analyzed in the following predefined subgroups: age, sex, body mass index, qualifying event, previous hypertension, previous ischemic stroke or TIA, and current smoker. The Cox proportional hazards model was fitted to each subgroup separately by modeling the primary outcome with treatment, subgroup, and treatment-by-subgroup interaction terms. Full methods are in the statistical analysis plan (Supplement 2). Statistical analyses were performed using the SAS, version 9.4 (SAS Institute).

### Early Study Termination

Study enrollment was terminated on December 31, 2023, by the steering committee before database lock and without unmasking due to lower-than-expected enrollment and financial limitations. The trial was funded entirely by government grants and received no support from any third-party for-profit organizations. The sponsor and the funder played no roles in this decision. The data monitoring committee was informed only after the decision had been made. Due to the early termination of enrollment, the trial did not achieve its planned recruitment schedule or target sample size. All enrolled patients completed protocol-defined follow-up for 90 days. Given the reduced sample, a post hoc power analysis was performed using final stroke recurrence rates (7.9% liraglutide vs 13.8% control), yielding a statistical power of 67% (Gpower, version 3.1.9.7).

## Results

### Trial Population

**Figure 1.**
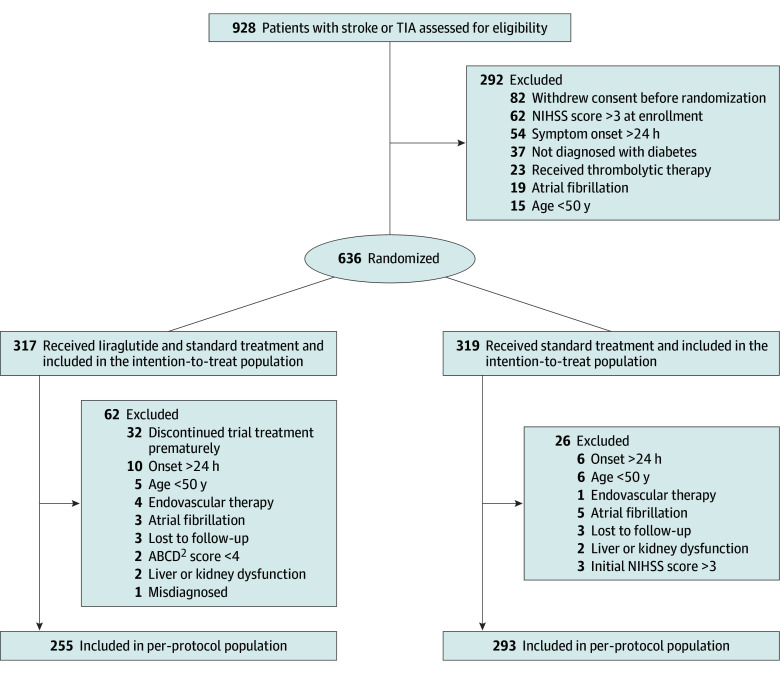
Enrollment and Randomization of Patients ABCD^2^, age, blood pressure elevation on first assessment after transient ischemic attack, unilateral weakness, speech disturbance, duration of symptoms and diabetes; NIHSS, National Institutes of Health Stroke Scale; TIA, transient ischemic attack.

A total of 928 patients were screened across 27 sites, and 636 (68.5%) were randomized for ITT analysis (Figure 1). Of these, 317 patients (49.8%) received standard treatment plus liraglutide, and 319 patients (50.2%) received standard treatment alone. In the liraglutide group, 32 patients (10.1%) did not adhere to the prescribed use of liraglutide (defined as discontinuing the medication for >20% of the study duration). The proportions of patients with ischemic stroke were 95.9% and 97.5% in the liraglutide and control groups, respectively. Baseline patient characteristics were comparable between groups (Table 1). The median (IQR) age of the overall sample was 63.5 (57.8-70.0) years, 36.3% were women, and 90.7% had a history of diabetes. The baseline glycated hemoglobin A_1c_ level was 8.2% (to convert to the total proportion of hemoglobin, multiply by 0.01). Six patients (0.9%) were lost to follow-up at 90 days. Details regarding medications used for the secondary prevention of ischemic stroke are provided in Table 1. Both groups maintained glycemic control within the guideline-recommended range. Information on glycemic control and hypoglycemic medication use are provided in eTable 4 in Supplement 3. Additional details of the per-protocol analysis are provided in eFigures 1, 3, and 4 and eTables 3 and 5 in Supplement 3.

**Table 1.** Patient Baseline Characteristics in the Intention-to-Treat Groups^a^

Characteristic	No. (%)	
	Control (n = 319)	Liraglutide (n = 317)
Age, median (IQR), y	64.0 (59.0-70.0)	63.0 (57.0-70.0)
Sex		
Female	115 (36.1)	116 (36.6)
Male	204 (63.9)	201 (63.4)
BMI, median (IQR)	23.7 (22.0-26.0)	24.2 (22.5-26.0)
Random blood glucose on admission, median (IQR), mg/dL	205.4 (149.9-287.8)	212.6 (144.1-263.1)
Hemoglobin A_1c_ level on admission, median (IQR), %	8.2 (6.8-10.0)	8.1 (7.0-9.8)
Medical history		
Diabetes^b^	287 (90.0)	290 (91.5)
Hypertension	222 (69.6)	230 (72.6)
Dyslipidemia	40 (12.5)	48 (15.1)
Previous ischemic stroke	91 (28.5)	80 (25.2)
Previous TIA	10 (3.1)	10 (3.2)
Coronary heart disease	27 (8.5)	30 (9.5)
Median blood pressure, mm Hg		
Systolic	154.0 (140.0-166.0)	152.0 (139.0-167.0)
Diastolic, mean (SD)	87.4 (13.1)	89.1 (12.0)
Current smoker	100 (31.3)	110 (34.7)
Qualifying event		
Stroke	311 (97.5)	303 (95.9)
TIA	8 (2.5)	13 (4.1)
TOAST classification^c^		
Large-artery atherosclerosis	152 (48.9)	146 (48.2)
Cardioembolic^d^	5 (1.6)	3 (1.0)
Small-vessel occlusion	144 (46.3)	145 (47.9)
Stroke of other determined etiology	0	0
Stroke of undetermined etiology	10 (3.2)	9 (3.0)
NIHSS score in patients with qualifying ischemic stroke, median (IQR)^e^	2.0 (1.0-3.0)	2.0 (1.0-2.0)
ABCD^2^ score in patients with qualifying TIA, median (IQR)^f^	4.0 (4.0-5.0)	5.0 (4.0-5.0)
Antithrombotic therapy		
Dual antiplatelet therapy	250 (78.4)	248 (78.2)
Single antiplatelet therapy	62 (19.4)	63 (19.9)
Anticoagulant therapy	2 (0.6)	4 (1.3)
No	5 (1.6)	2 (0.6)
Lipid-lowering therapy		
Yes	308 (96.6)	306 (96.5)
No	11 (3.4)	11 (3.5)
Antihypertensive therapy		
Yes	207 (64.9)	216 (68.1)
No	112 (35.1)	101 (31.9)

Abbreviations: ABCD^2^, age, blood pressure elevation on first assessment after transient ischemic attack, unilateral weakness, speech disturbance, duration of symptoms and diabetes; BMI, body mass index (calculated as weight in kilograms divided by height in meters squared); NIHSS, National Institutes of Health Stroke Scale; TIA, transient ischemic attack; TOAST, Trial of ORG 10172 in Acute Stroke Treatment.

SI conversion factors: to convert glucose to mmol/L, multiply by 0.0555; for hemoglobin A_1c_ to the proportional of total hemoglobin, multiply by 0.01.

^a^ There were no significant differences between the treatment groups for any characteristic.

^b^ These patients had a history of type 2 diabetes, while the rest had a new diagnosis of diabetes.

^c^ The presumed stroke cause was classified according to TOAST criteria using clinical findings, brain imaging results, and laboratory test results. A total of 21 patients with TIA and 1 patient with myelitis lacked TOAST classification information.

^d^ These patients did not have a documented history of cardiogenic stroke at enrollment but were later identified as having cardioembolic stroke during their hospital stay.

^e^ NIHSS is a standardized neurologic examination comprising 15 questions covering 11 specific functions scored on a scale of 0 to 4, in which 0 indicates normal functioning and 4 indicates complete impairment; a score of 42 indicates death.

^f^ Among patients with TIA, the qualifying score was 4 or more on the ABCD^2^ scale, which ranges from 0 to 7, with higher scores indicating a greater risk of stroke. The scale is used to estimate the risk of recurrent stroke after a TIA based on age, blood pressure, clinical features, duration of symptoms, and presence of diabetes.

### Primary Outcome

**Figure 2.**
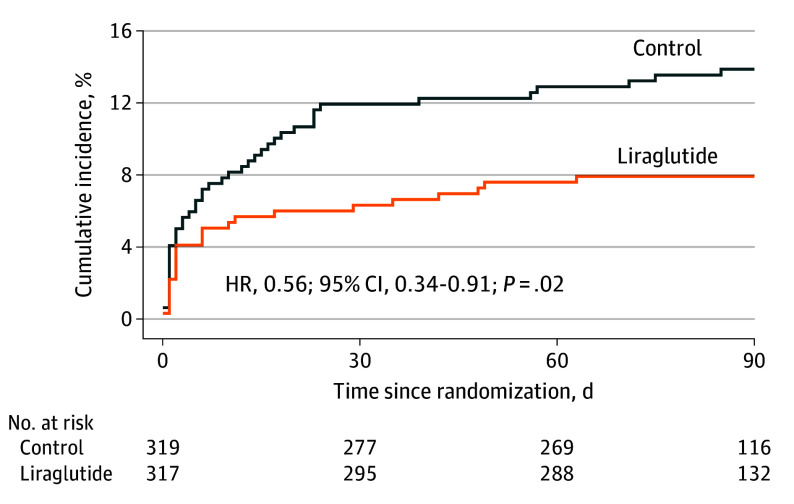
Cumulative Incidence of Stroke Primary outcome. HR indicates hazard ratio.

In total, 25 primary outcome events (new ischemic or hemorrhagic strokes within 90 days) were observed in the liraglutide group (7.9% of 317 patients) and 44 (13.8% of 319 patients) in the control group. In the ITT population, compared with the control condition, liraglutide treatment significantly reduced the risk of stroke recurrence (HR, 0.56; 95% CI, 0.34-0.91; *P* = .02) (Figure 2 and Table 2).

**Table 2.** Trial Outcomes in the Intention-to-Treat Group^a^

Outcome	Control (n = 319)		Liraglutide (n = 317)		Treatment effect, HR (95% CI) ^a^	*P* value
	Patients with event, No.	Incidence, %	Patients with event, No.	Incidence, %		
Primary outcome						
Stroke^b^	44	13.8	25	7.9	0.56 (0.34-0.91)	.02
Ischemic stroke	43	13.5	24	7.6	0.55 (0.33-0.90)	
Hemorrhagic stroke	1	0.3	1	0.3	0.96 (0.06-15.31)	
Secondary outcomes						
Vascular event within 90 d^c^	50	15.7	27	8.5	0.53 (0.33-0.84)	.01
mRS score^d^^,^^e^						NA
≤1	246	77.8	274	87.3	OR,1.95 (1.28-3.00)	.002
					RR,1.12 (1.04-1.21)	.002
≤2	274	86.7	289	92.0	OR,1.77 (1.06-3.02)	.03
					RR,1.06 (1.01-1.12)	.03
Safety outcomes						
sICH^f^	2	0.6	1	0.3	0.48 (0.04-5.27)	.50
Hypoglycemia^g^	25	7.8	24	7.6	0.93 (0.53-1.62)	.80
Gastrointestinal disorders^h^	13	4.1	60	18.9	4.81 (2.64-8.78)	<.001
Pneumonia	2	0.6	2	0.6	0.97 (0.14-6.90)	>.99
Acute pancreatitis	0	0	0	0	NA	NA
Death^i^	4	1.3	1	0.3	0.24 (0.03-2.10)	.10

Abbreviations: HR, hazard ratio; mRS, modified Rankin Scale; NA, not applicable; OR, odds ratio; RR, risk ratio; sICH, symptomatic intracerebral hemorrhage.

^a^ The common OR/RR is shown for mRS. HRs were analyzed with the use of a Cox proportional hazards model with treatment as a categorical fixed factor. ORs were calculated using a generalized linear model. RRs were calculated using modified Poisson regression model.

^b^ Ischemic stroke and hemorrhagic stroke are presented as subtypes of the primary outcome (stroke at 90 days).

^c^ Vascular events included ischemic stroke, hemorrhagic stroke, transient ischemic attack, myocardial infarction, and vascular death.

^d^ The mRS is a 6-point scale used to measure the degree of global disability, with scores ranging from 0 (no symptoms) to 6 (death), in which 1 indicates no significant disability and 5 indicates severe disability. An mRS score of 0 or 1 is considered excellent.

^e^ Three patients were lost to follow-up in the liraglutide control groups.

^f^ sICH based on the European Cooperative Acute Stroke Study II definition.

^g^ Hypoglycemic events were defined as blood glucose levels less than 70 mg/dL (to convert to mmol/L, multiply by 0.0555).

^h^ Gastrointestinal disorders included nausea, vomiting, diarrhea, abdominal pain, and dyspepsia.

^i^ Death denoted the all-cause mortality rate at 90 days.

### Secondary Outcomes

For secondary outcomes, new clinical vascular events within 90 days occurred for 27 of the 317 patients (8.5%) in the liraglutide group and 50 of the 319 patients (15.7%) in the control group (HR, 0.53; 95% CI, 0.33-0.84; *P* = .01). The percentage of patients who achieved an excellent functional outcome (mRS score ≤1) at 90 days was 274 of 314 (87.3%) in the liraglutide group and 246 of 316 (77.8%) in the control group. In the ITT population, patients receiving liraglutide had significantly greater odds of achieving an excellent outcome than controls (OR, 1.95; 95% CI, 1.28-3.00; *P* = .002). Similarly, 289 of 314 patients (92.0%) in the liraglutide group achieved a favorable functional outcome (mRS score ≤2) at 90 days compared with 274 of 316 (86.7%) in the control group. The odds of achieving a favorable outcome were also significantly higher in the liraglutide group (OR, 1.77; 95% CI, 1.06-3.02; *P* = .03) (Table 2). Groupwise distribution of mRS scores is provided in eFigure 2 of Supplement 3.

### Safety Outcomes

The safety outcome of sICH was observed in 1 of 317 patients (0.3%) in the liraglutide group compared with 2 patients (0.6%) in the control group (HR, 0.48; 95% CI, 0.04–5.27; *P* = .5). Hypoglycemia occurred in 24 patients (7.6%) in the liraglutide group and 25 patients (7.8%) in the control group (HR, 0.93; 95% CI, 0.53-1.62; *P* = .80). Gastrointestinal disorders were reported in 60 patients (18.9%) who received liraglutide and only 13 patients (4.1%) in the control group (HR, 4.81; 95% CI, 2.64-8.78; *P* < .001). No significant differences were observed between groups in the incidence of pancreatitis, pneumonia, or all-cause mortality (Table 2).

### Subgroup Analysis

**Figure 3.**
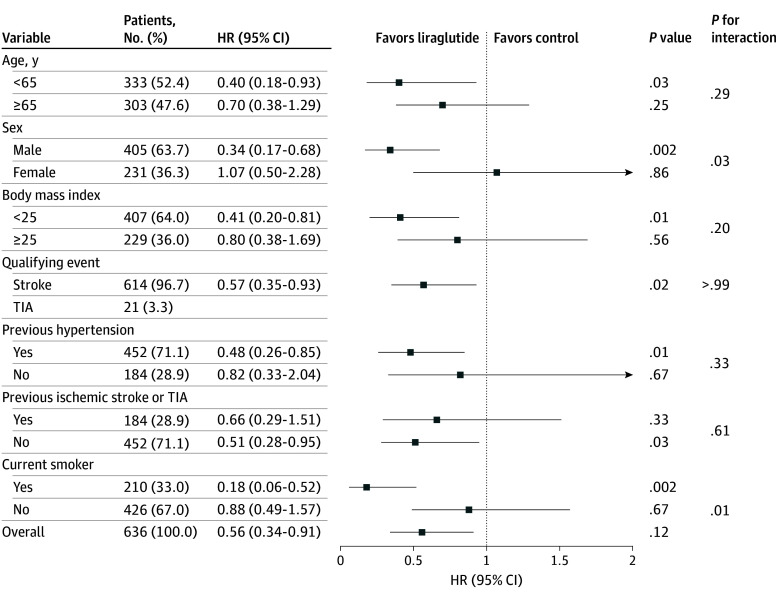
Hazard Ratios (HRs) for Recurrent Stroke at 90 Days (Primary Outcome) in Prespecified Subgroups A Cox proportional hazards model was fitted to each subgroup separately by modeling the primary outcome with treatment, subgroup, and treatment-by-subgroup interaction terms (body mass index calculated as weight in kilograms divided by height in meters squared). The primary outcome was the percentage of patients who developed a stroke recurrence (ischemic or hemorrhagic stroke) within 90 days. For subcategories, squares represent point estimates (with the area of the square proportional to the number of events) and horizontal lines represent the 95% CI. TIA indicates transient ischemic attack.

The results of the subgroup analysis for the primary outcome are presented in Figure 3. The effects of liraglutide were more pronounced among those who smoked and male participants, suggesting that smoking status and sex influenced the efficacy of the investigational drug.

## Discussion

In this multicenter randomized clinical trial, patients with T2D who had experienced minor AIS or high-risk TIA were assigned to receive basic therapy plus liraglutide or basic therapy within 24 hours of symptom onset. Patients receiving liraglutide exhibited a lower risk of recurrent stroke and a higher rate of excellent functional outcomes (mRS score ≤1) at 90 days without an increased incidence of symptomatic intracranial hemorrhage or mortality. Consistent with prior studies, the stroke recurrence rate and 90-day neurological outcomes observed in the control group closely mirrored those reported in similar patient populations, thereby strengthening the external validity of the present findings.^5,21^ To our knowledge, this is the first phase 3 randomized clinical trial to evaluate the efficacy and safety of liraglutide in patients with minor AIS or high-risk TIA and provide preliminary evidence that supports its potential role in secondary stroke prevention.

Although liraglutide was associated with a favorable HR (0.56), this result should be interpreted cautiously, given the relatively low number of primary end point events (n = 69). While these findings suggest a promising signal, they require further validation in larger, adequately powered trials.

The Kaplan-Meier curves in our study showed a difference in recurrence risk between the 2 groups that emerged within the first month after randomization, suggesting a possible early benefit. This early effect may be attributed to several interrelated mechanisms. First, liraglutide significantly improves insulin sensitivity within 2 weeks of initiation,^22^ potentially correcting acute-phase metabolic disturbances in patients with T2D and AIS. Supporting this, the IRIS trial demonstrated that improving insulin resistance can reduce the risk of stroke recurrence, although the trial enrolled individuals without diabetes.^23^ Second, GLP-1RAs exhibit potent vascular anti-inflammatory effects by modulating macrophage polarization, suppressing nuclear factor κ B activation, and reducing monocyte-endothelial adhesion while simultaneously enhancing endothelial function through upregulation of endothelial nitric oxide synthase, restoration of prostacyclin activity, and reduction of oxidative stress within vascular endothelial cells.^24,25,26^ Experimentally, GLP-1RAs inhibit platelet aggregation by activating platelet cyclic adenosine monophosphate and endothelial nitric oxide synthase signaling, thereby reducing thrombus formation.^27^ Together, these mechanisms may contribute to the observed reduction in early stroke recurrence. In addition to these early effects, GLP-1RAs attenuate atherosclerosis progression,^14^ which may contribute to sustained vascular protection over the long term.

These biological mechanisms are partially supported by prior clinical evidence. A large-scale meta-analysis established the role of GLP-1RAs in the primary prevention of stroke.^28^ Our findings contribute evidence to this field, suggesting that early administration of GLP-1RAs may confer clinical benefit in patients with T2D who are experiencing minor AIS or high-risk TIA.

In subgroup analyses, the effects of liraglutide appeared more pronounced among people who smoked and male participants, which is potentially hypothesis generating. These subgroups may have been associated with higher levels of systemic inflammation and insulin resistance, which could potentiate the metabolic and anti-inflammatory effects of GLP-1RA therapy.^29^ However, our sample size did not reach the original target, possibly resulting in insufficient power to reliably detect interaction effects. Further, previous cardiovascular outcome trials involving GLP-1RAs have not consistently shown similar subgroup effects. Therefore, these findings should be cautiously interpreted.

Although weekly formulations of semaglutide and dulaglutide are considered convenient drugs for patients with T2D, direct evidence on these drugs being more effective than liraglutide in reducing stroke risk is lacking. In this article, we opted for daily liraglutide administration because of concerns that persistent hiccups or vomiting might increase the risk of pneumonia in patients with acute stroke, especially among those experiencing severe gastrointestinal reactions. Short-acting medications allow for prompt dose adjustment or discontinuation as needed, which may help mitigate this risk. Nevertheless, we reported a relatively high dropout rate in the liraglutide group, possibly due to gastrointestinal adverse events. Specifically, 18.9% of patients receiving liraglutide experienced gastrointestinal disorders, such as nausea, vomiting, and diarrhea, that led to treatment discontinuation in some cases. Moreover, the injection method and administration frequency posed challenges for certain patients, further contributing to nonadherence.

### Limitations

Our study had limitations. First, the early termination of the trial introduced uncertainty into the observations and increased the risk of failing to detect a true treatment effect. Although a treatment effect was observed, its external validity and generalizability to broader populations remain uncertain. Second, the possibility of information bias cannot be ignored because the assigned treatment was not concealed by participants or physicians. However, to minimize measurement bias and ensure the objective measurement of the primary end point, we used masked end point assessments. Third, the generalizability of the current results is limited by the exclusion criteria, in which patients who did not have T2D, those with ischemic stroke (National Institutes of Health Stroke Scale score >3), individuals with stroke of cardiac origin, patients who initiated treatment later than 24 hours after symptom onset, and patients who were undergoing or planning to undergo thrombolysis or thrombectomy were excluded. Fourth, since the LAMP study only included Chinese patients, the findings may not apply to other populations. Fifth, our prespecified protocol did not entail collecting standardized laboratory-based follow-up data on hemoglobin A_1c_ levels or body mass index at 90 days. Therefore, we were unable to explore whether improvements in long-term glycemic control or weight reduction contributed to the observed treatment effects. Sixth, the original sample size estimation was based on a simplified binary approach rather than a formal time-to-event model, potentially leading to imprecise assumptions regarding statistical power.

## Conclusions

The results of this randomized clinical trial suggest that, among Chinese patients with minor AIS or high-risk TIA who received diagnosis of T2D, treatment with liraglutide might be associated with a lower risk of recurrent stroke and improved 90-day outcomes. However, given the early termination of the trial, small sample size, and limited number of outcome events, the possibility that these findings were due to chance cannot be excluded. Larger, adequately powered studies are needed to confirm the observed effects and establish their reliability.

## Notes

Supplement 1. Trial protocol

Supplement 2. Statistical analysis plan

Supplement 3. eAppendix 1. Recruitment by Site in LAMP Trial eAppendix 2. Committee Members eMethods. eFigure 1. Trial Profile eFigure 2. Distribution of Modified Rankin Scale Scores at 90 Days in the Intention-to-Treat eFigure 3. Distribution of Modified Rankin Scale Scores at 90 Days in the Per-Protocol Analysis eFigure 4. Primary Outcome by Prespecified Subgroups in the Per-Protocol Analysis eTable 1. Criteria for the diagnosis of diabetes eTable 2. Definitions of stroke events and vascular events eTable 3. Baseline Characteristics of the Population in the Per-Protocol Analysis eTable 4. The patient's glycemic control and antidiabetic drug use status in Full Analysis Set eTable 5. Trial Outcomes in the Per-Protocol Analysis

Supplement 4. Nonauthors collaborators

Supplement 5. Data sharing statement
