# Supplementary material for: Liraglutide in Acute Minor Ischemic Stroke or High-Risk Transient Ischemic Attack With Type 2 Diabetes: The LAMP Randomized Clinical Trial
Source: JAMA Intern Med. 2025 Nov 3;186(1):46–54. doi: 10.1001/jamainternmed.2025.5684 (PMC12584062; doi:10.1001/jamainternmed.2025.5684)
Supplement: Supplement 1. — Trial protocol [file jamainternmed-e255684-s001.pdf]

PROTOCOL

Version 1.0 of 2019

Liraglutide in Acute Minor Ischemic Stroke or High-risk Transient Ischemic Attack Patients with Type 2 Diabetes Mellitus (LAMP): A Multicenter, Controlled, Prospective, Randomized, Open-label, Blinded Endpoint (PROBE) Trial

Principle Investigator:
Anding Xu, Huili Zhu
Department of Neurology
the First Affiliated Hospital of Jinan University
Guangzhou, 510000, China
Tel: +8613392692160, +8613922165588
E-mail: tlil@jnu.edu.cn, zhlffff@163.com

Table of Contents

Abstract....4
Abbreviation....7
1. Background and current state of knowledge....8
2. Objectives of the trial....8
3. Design and selection of patients....8
3.1 Trail plan....8
3.2 Selection criteria....8
3.3 Suspension Criteria:....9
3.3 Duration of participation for each subject....9
3.4 Randomization and Masking....9
4. Plan and conduct of the trial....9
4.1 Clinical trial flow chart....9
4.2 Study completion....10
4.3 Study termination....10
5. Study medications....10
5.1 Identification of study drugs....10
5.2 Responsibilities of study drugs administration....10
6. Treatment administered to included subjects....10
6.1 Description of the treatment required to conduct the study....10
6.2 Permitted and prohibited medical drugs and treatments in the study....10
7. Outcome measurements....10
7.1 Primary efficacy outcome....10
7.2 Secondary efficacy outcomes....11
7.3 Safety outcomes....11
7.4 Study flow diagram....11
7.5 Study periods....11
8. Description of safety assessment parameters....12
8.1 Evaluate clinical safety....12
8.2 Safety assessment....12
8.3 Safety report....14
9. Statistics....15
9.1 Sample size....15
9.2 Statistical analysis plan....15

9.3 Analysis population 15
10. Study organization 16
11. Data management and monitoring 16
11.1 Training of study site personnel 16
11.2 Monitoring of the study 16
Appendix 17
Appendix 1: Definitions of stroke events and vascular events 17
Appendix 2: Criteria for the diagnosis of diabetes[14] 20
Appendix 3: National Institutes of Health Stroke Scale (NIHSS)[15] 21
Appendix 4: Modified Rankin Score (mRS) 26
Appendix 5: Montreal Cognitive Assessment (MoCA)[16] 27
Reference 28

Abstract

**Title**	Liraglutide in Acute Minor Ischemic Stroke or High-risk Transient Ischemic Attack Patients with Type 2 Diabetes Mellitus (LAMP): A Multicenter, Controlled, Prospective, Randomized, Open-label, Blinded Endpoint (PROBE) Trial
**Principle Center**	The First Affiliated Hospital of Jinan University
**Objective**	To investigate the safety and efficacy of the Glucagon-like peptide-1 (GLP-1) receptor agonists liraglutide in treating acute mild ischemic stroke (National Institutes of Health Stroke Scale [NIHSS] score ≤3) or high-risk transient ischemic attack (TIA) (ABCD2 score ≥4) patients with type 2 diabetes mellitus.
**Efficacy Outcomes**	Primary outcome:<br>1. The proportion of patients who experience stroke recurrence (ischemic or hemorrhagic) within 90 ± 7 days.<br>Secondary outcome:<br>1. The percentage of patients experiencing new clinical vascular events at 90 ± 7 days, including ischemic stroke, hemorrhagic stroke, TIA, myocardial infarction, and vascular death.<br>2. The proportion of patients achieving a modified Rankin Scale (mRS) score ≤1 or ≤2 points and evaluate changes in mRS scores at 90 ± 7 days.<br>Exploratory outcome:<br>1. Changes in cognitive status (Montreal Cognitive Assessment [MoCA] score) on day 90 ± 7.
**Safety Outcomes**	1. The rate of symptomatic intracerebral hemorrhage at 90±7 days.<br>2. The proportion of pancreatitis at 90 ± 7 days.<br>3. The percentage of hypoglycemic events (blood glucose <3.9 mmol/L) at 90 ± 7 days.<br>4. The percentage of gastrointestinal disorders events at 90 ± 7 days.<br>5. The percentage of pneumonia at 90 ± 7 days.<br>6. 90-day all-cause mortality.<br>7. Adverse events, and severe adverse events through 90 ± 7 days of follow-up.
**Trial Design**	LAMP is a Multicenter, Controlled, Prospective, Randomized, Open-label, Blinded Endpoint (PROBE) Trial to assess liraglutide's safety and effectiveness in reducing stroke recurrence and improving prognosis for acute mild ischemic stroke/ high-risk TIA patient with type 2 diabetes mellitus. Follow-up data were collected on day 7 ± 1, 30 ± 3 days, and 90 ± 7 days after randomization. All the outcome assessors were masked to the allocation assignment and follow-up.
**Trial Population**	Acute mild ischemic stroke (NIHSS score ≤3) or high-risk transient ischemic attack (ABCD2 score ≥4) patients with type 2 diabetes mellitus.
**Sample Size**	1708
**Inclusion criteria**	1. Adult patients (male or female, ≥ 50 years).<br>2. Acute ischemic stroke (NIHSS ≤3 at the time of randomization) or high-risk transient ischemic attack (ABCD2 ≥ 4 at the time of randomization) patients with type 2 diabetes mellitus within 24 hours of symptom onset.<br>3. First stroke or prior stroke without sequel (modified Rankin Scale ≤1).<br>4. Informed consent signed.
**Exclusion criteria**	1. Diagnosis of intracranial hemorrhagic diseases on baseline computed tomography.<br>2. Iatrogenic or cardiogenic stroke.<br>3. Patients undergo thrombolysis or endovascular treatment.

	4. Regular GLP-1 receptor agonists used in the last 90 days.<br>5. Family/personal history of multiple endocrine neoplasia type 2 or familial medullary thyroid carcinoma.<br>6. Patients with pancreatitis or previous history of pancreatitis, inflammatory bowel disease, or gastroparesis.<br>7. Pregnant, lactating women, or patients likely or planning to become pregnant.<br>8. Allergic to liraglutide or excipients.<br>9. Congestive heart failure (New York Heart Association class III-IV).<br>10. Severe liver or kidney dysfunction (aspartate transaminase/alanine transaminase ratio or serum creatinine are 3 times higher than the normal upper limit).<br>11. Participated in other clinical trials of drugs within 90 days.<br>12. Patients who are ineligible to participate in this clinical study are deemed by researchers.
**Trial Cycle**	All included patients were followed up at baseline, 7 ± 1 days, 30 ± 3 days, and 90 ± 7 days after randomization, respectively.
**Treatment Regimens**	In the treatment arm, the initial dosage of liraglutide was 0.6 mg per day, which subsequently increased to 1.2 mg per day in the second week and further escalated to 1.8 mg per day in the third week through subcutaneous injection once daily. From then on, a consistent dosage of 1.8 mg per day was maintained until day 90. The control arm will not utilize liraglutide. Both arms are prohibited from using other types of GLP-1RA, dipeptidyl peptidase-IV inhibitors, or sodium-glucose cotransporter-2 inhibitors.
**Procedure**	**Screening period**: On day 0 (baseline period), it is necessary to complete enrolment screening, and collect demographic characteristics, medical history (including the history of hypertension, diabetes, and drug treatment history, etc.), neurological measurements (NIHSS score, mRS score), haematological examination (blood routine, blood glucose, hepatic and renal function, serum amylase, insulin, C-peptide, high-sensitivity C-reactive protein, etc.) and other information.<br>**Treatment period**: the control group receives standard treatment, and the treatment group receives standard treatment plus liraglutide.<br>**Follow-up period**: NIHSS score was assessed at baseline, 7 ±1 days, and 90 ± 7 days after randomization. The mRS score was assessed at baseline, 30 ± 3 days, and 90 ± 7 after randomization. All concomitant medications, adverse events, stroke recurrence, and other vascular events of each visit were recorded since the last visit.<br>All the adverse events of included subjects should be recorded and tracked until properly resolved.<br>All the serious adverse events of included subjects should be recorded and tracked, even if the subjects have finished the trial, until the events were resolved, or stabilization judged by the investigator.
**Concomitant Treatment**	Guideline-based treatment (2014 China Secondary Prevention Guideline for Ischemic Stroke and Transient Ischemic Attack as well as the 2017 China Diabetes Prevention Guideline[1,2])

**Statistical Analysis**	Efficacy and safety analyses were performed in the intention-to-treat population. Kaplan-Meier will be used to estimate the cumulative stroke risk (ischemic or hemorrhagic) for the 90-day follow-up, with hazard ratios and 95% confidence interval using Cox proportional hazards methods and the log-rank test to evaluate the treatment effect, with trial centers included as a random effect. When multiple events of the same type occur, the time to the first event is utilized. Patients who do not have a primary outcome are censored at the time of death, last known contact, or at 90 days, whichever comes first. Data before drug administration will be the baseline data. Similar approaches were used for comparison of the secondary outcomes of clinical vascular events and comparison of safety outcomes. Measurement data between the two groups will be compared using two independent sample t-tests and mean ± standard deviation will be used for statistical description. If the data distribution is non-normal, we will use Mann–Whitney U test tests. The Chi-square test and Fisher exact test will be used to compare the counting data between the two groups, and percentages will be used for statistical description.<br>The proportion of mRS 0-1 at 90 ± 7 days and mRS 0-2 at 90 ± 7 days between the two groups will be compared through binary logistic analysis with odds ratios and 95% confidence interval. P-value<0.05 will indicate statistical significance. Statistical analyses will be performed with SAS software version 9.4 (SAS Institute).
**Sites Number**	About 30
**Duration**	36 months

99 # Abbreviation

Abbreviation	Full title
AE	Adverse Event
AIS	Acute Ischemic Stroke
ALT	Alanine Aminotransferase
AST	Aspartate Transaminase
APTT	Activated Partial Thromboplastin Time
BUN	Blood Urea Nitrogen
Cr	Creatinine
CRF	Clinical Research Form
CT	Computed Tomography
GCP	Good Clinical Practice
GLU	Glucose
GLP-1	Glucagon-like peptide-1
GLP-1RA	GLP-1 receptor agonists
IQR	Inter-Quartile Range
ITT	Intention to Treat
LAA	large-artery atherosclerosis
MRI	Magnetic Resonance Imaging
mRS	Modified Rankin Scale
NIHSS	National Institute of Health Stroke Scale
PPS	Per Protocol Set
PT	Prothrombin Time
rtPA	Recombinant tissue-type plasminogen activator
SAE	Serious Adverse Event
SD	Standard Deviation
SS	Safety Set
TC	Total Cholesterol
TG	Triglyceride
TOAST	Trial of Org 10 172 in acute stroke treatment
T2DM	Type 2 diabetes mellitus

1. Background and current state of knowledge

Type 2 diabetes mellitus (T2DM) is identified as one of the primary risk factors for ischemic cerebrovascular disease, about one-third of stroke patients have diabetes mellitus[3-5]. Approximately 4% to 10% of patients with acute minor ischemic stroke or TIA may experience a recurrent stroke or TIA within 90 days [6-8]. Patients with T2DM who have experienced a minor ischemic stroke or TIA are more susceptible to stroke recurrence compared to those with normal glucose levels, particularly in cases of the subtype of stroke caused by large-artery atherosclerosis(LAA)[9,10]. The protective role of Glucagon-like peptide-1 receptor agonists (GLP-1RA) in cardiovascular events has been demonstrated[11,12]. However, it remains unclear whether GLP-1RA has a protective effect on acute ischemic stroke and TIA patients.

2. Objectives of the trial

The objective of this study is to investigate the safety and efficacy of the GLP-1 receptor agonists (GLP-1RA) liraglutide in treating acute mild ischemic stroke (NIHSS score ≤3) or high-risk transient ischemic attack (ABCD2 score ≥4) patients with type 2 diabetes mellitus (not only those with a previous history of diabetes, but also newly diagnosed diabetic patients).

3. Design and selection of patients

3.1 Trail plan

The LAMP trial is a Multicenter, Controlled, Prospective, Randomized, Open-label, Blinded Endpoint (PROBE) Trial with a duration of 90 days. Please refer to Figure 1 for the detailed study design. Recruitment commenced in June 2019 and finished in December 2023. The study was registered with ClinicalTrials.gov (ID: NCT03948347).

3.2 Selection criteria

Table 1. Inclusion criteria

Adult patients (male or female, ≥50 years)
Acute ischemic stroke (NIHSS ≤3 at the time of randomization) or high-risk transient ischemic attack ($ABCD^2$ ≥4 at the time of randomization) patients with type 2 diabetes mellitus within 24 hours of symptom onset
First stroke or prior stroke without sequel (modified Rankin Scale ≤1)
Informed consent signed

Table 2. Exclusion criteria

Diagnosis of intracranial hemorrhagic diseases on baseline computed tomography
Iatrogenic and cardiogenic stroke
Patients undergoing thrombolysis or endovascular treatment
Regular glucagon-like peptide-1 analogue use in last 90 days
Family/personal history of multiple endocrine neoplasia type 2 or familial medullary thyroid carcinoma
Patients with pancreatitis or previous history of pancreatitis, inflammatory bowel disease, and gastroparesis
Pregnant, lactating women, or patients likely or planning to become pregnant
Allergic to liraglutide or excipients
Congestive heart failure (New York Heart Association class III-IV)
Severe liver or kidney dysfunction (aspartate transaminase/alanine transaminase ratio and serum creatinine are 3 times higher than normal upper limit)
Patients with malignant tumors expected to survive for < 3 months
Participated in other clinical trials of drugs within 3 months

Patients who are ineligible to participate in this clinical study deemed by researchers

3.3 Suspension Criteria:

Trial suspension means that the clinical trial has not finished as planned, and all trials are stopped in the middle period. The purpose of trial suspension is to protect the rights and interests of subjects, ensure the quality of the trial, and avoid unnecessary economic losses:

1. The proportion of serious adverse events during the trial is higher than 10%, and the Data Monitoring Committee (DMC) has the right to terminate the study unconditionally.
2. Trial should be discontinued when one treatment is found to be significantly better than the other

3.3 Duration of participation for each subject

Each subject will be followed up in the trial for 90 days.

3.4 Randomization and Masking

Randomization is allowed after obtaining written informed consent. Patients will be randomly assigned to the liraglutide treatment group and the control group according to the block random method using sealed envelopes (1:1 ratio). Only those responsible for evaluating the results were unaware of the allocation.

4. Plan and conduct of the trial

4.1 Clinical trial flow chart

Tria procedure / items	1st follow-up[1]	2st follow-up[1]	3st follow-up[1,2]	4st follow-up[1,3,4]
	Day1	Day7±1	Day 30±3	Day 90±7
Sign informed Consent	√			
Identify inclusion/ exclusion criteria	√			
Medical history, family history, and life history	√			
Characteristics of Patients	√			
Physical examination	√			
NIHSS score	√	√		√[6]
MOCA score	√			√[6]
mRS score	√		√	√
TOAST subtype		√		
Laboratory examination	√	√[5]		
The use of Liraglutide in study		√	√	√
Concomitant medications	√	√	√	√
AE、SAE、outcomes	√	√	√	√

1. The drug used in the liraglutide treatment group must be strictly followed for 90 days, and normal discharged patients are required to take the medicine home and continue taking it for 90 days.

2. Visit 3 is followed by a third-party telephone visit, and the research team does not participate in this visit.
3. Visit 4 is followed up by a third-party telephone or in-person visit, and the research team does not participate in this visit. It is recommended that all patients undergo an in-person visit.
4. If the trial is terminated before Visit 4, a visit must also be conducted at the next visit point.
5. For patients with a hospital stay of at least 7 days, the most recent laboratory test results on the day closest to Day 7 are recorded; for patients with a hospital stay of less than 7 days, the most recent laboratory test results on the day closest to the discharge date are recorded.
6. For patients who undergo an in-person visit at Visit 4, the NIHSS score and MoCA score must be recorded.

4.2 Study completion

The study is considered to be finished when the last visit of the last subject in the trial is completed.

4.3 Study termination

The principal investigator has the right to close or suspend the study center at any time. The closure of the center should occur after all required documentation and study supplies have been collected, and a center closure visit has been conducted. Additionally, the principal investigator or investigator may close the center in advance if the investigator fails to comply with the requirements of the study protocol, IEC/IRB or local regulatory authorities, the principal investigator's operating procedures, or GCP guidelines.

5. Study medications

5.1 Identification of study drugs

All the trial drugs (Liraglutide) were purchased with research funds from the principal investigator. No confidentiality agreement existed between the authors and any commercial entity. Liraglutide is manufactured by manufactured by Novo Nordisk company.

5.2 Responsibilities of study drugs administration

The clinical trial investigator is accountable for ensuring the study drugs throughout the entire duration of the study.

The investigator of the clinical trial is responsible for ensuring that the study drugs received by the study centers are counted and recorded during the whole process of the study, and the distribution and recovery of the drug must be recorded in the Drug Dispensing and Recycling Record Form. The original packaging of the study drug should be returned, whether there is any remaining study drug in it. Returned study drugs should be kept at the site designated by the study center and separate from unused study drugs. Do not destroy or mix medicines in different packages until inspectors have counted them. The use of the study drug must strictly follow the instructions in the protocol and the package label. The principal Investigator monitor must be allowed to count and reconcile unused and returned study drugs at each monitoring. Unused or returned study drugs to be destroyed must be recorded on the Drug Recycling Record Form and returned to the medication destruction center.

6. Treatment administered to included subjects

6.1 Description of the treatment required to conduct the study

In the treatment arm, the initial dosage of liraglutide was 0.6 mg per day, which was subsequently increased to 1.2 mg per day in the second week and further escalated to 1.8 mg per day in the third week through subcutaneous injection once daily. From then on, a consistent dosage of 1.8 mg per day was maintained until day 90. If dose escalation results in unacceptable adverse effects, the intervals for dose escalation could be extended, treatment could be temporarily paused, or maintenance doses below the target dose of 1.8 mg per day could be considered. The control arm will not utilize liraglutide. Both arms received standard treatment according to the guidelines[1,2,13].

6.2 Permitted and prohibited medical drugs and treatments in the study

Both arms are prohibited from using other types of GLP-1RA, dipeptidyl peptidase-IV inhibitors, or sodium-glucose cotransporter-2 inhibitors.

7. Outcome measurements

7.1 Primary efficacy outcome

1. The proportion of patients who experience stroke recurrence (ischemic or hemorrhagic) within 90±7days.

The determination of stroke recurrence

The determination of whether new neurological deficits that occur after enrollment are attributable to stroke recurrence, stroke progression, or infarct growth often poses a challenge. The LAMP does not strictly

differentiate between recurrence and progression, as the likely pathophysiology of these events involves additional thrombosis[14]. We specifically defined stroke recurrence into the following categories and invited senior neurologists to identify stroke recurrence based on these terms.

Ischemic stroke: Events of neurological dysfunction resulting from a focal infarction in the brain, spinal cord, or retina. Any of the following criteria is indicative of a new ischemic stroke: 1) New focal neurological deficits lasting less than 24 hours, not attributable to non-ischemic causes, with neuroimaging evidence of a new cerebral infarction; 2) New focal neurological deficits lasting 24 hours or longer, with or without imaging evidence, and not attributable to non-ischemic causes.

Hemorrhagic stroke: Rapidly progressing neurological dysfunction symptoms caused by the accumulation of blood in the non-traumatic brain parenchyma, ventricular system, or subarachnoid space.

7.2 Secondary efficacy outcomes

1. The percentage of patients experiencing new clinical vascular events, including ischemic stroke, hemorrhagic stroke, transient ischemic attack (TIA), myocardial infarction, and vascular death at 90 ±7 days after randomization.
2. The proportion of patients achieving a modified Rankin Scale (mRS) score ≤2 or ≤1 points and evaluate changes in mRS scores at 90 ± 7 days after randomization.

7.3 Safety outcomes

1. The rate of symptomatic intracerebral hemorrhage at 90 ±7 days.
2. The proportion of pancreatitis at 90 ±7 days.
3. The percentage of hypoglycemic events (blood glucose <3.9 mmol/L) at 90 ± 7 days.
4. The percentage of gastrointestinal disorders events at 90 ± 7 days.
5. The percentage of pneumonia at 90 ±7 days.
6. 90-day all-cause mortality.
7. Adverse events, and severe adverse events through 90 ± 7 days of follow-up.

7.4 Study flow diagram

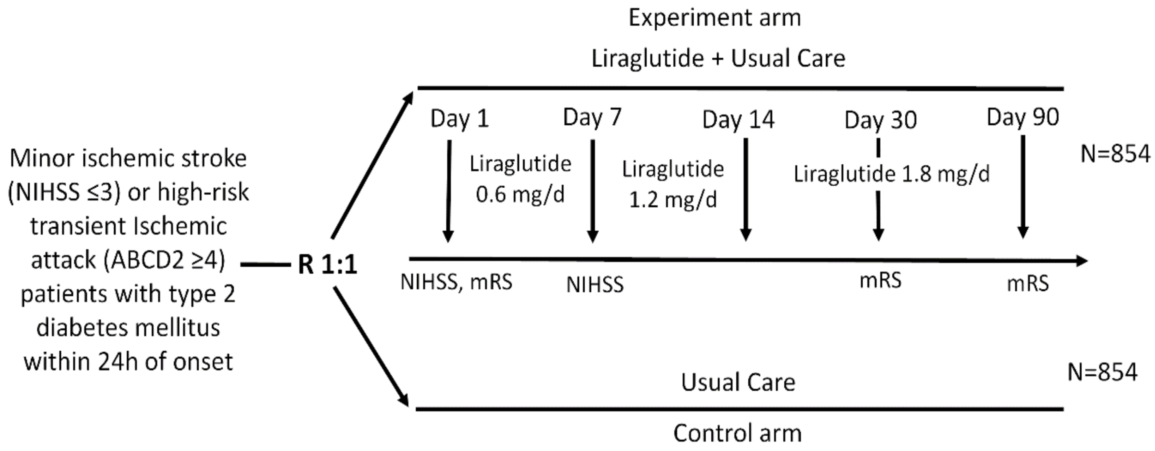

Fig. 1 Study flow diagram

7.5 Study periods

Screening period: On day 0 (baseline period), it is necessary to complete enrolment screening, and collect demographic characteristics, medical history (including the history of hypertension, diabetes, atrial fibrillation, and drug treatment history, etc.), neurological measurements (NIHSS score, mRS score), haematological examination (blood routine, blood glucose, hepatic and renal function, serum amylase, insulin, C-peptide, high-sensitivity C-reactive protein, etc.) and other information.

Treatment period: The control group received standard treatment, and the treatment group received standard treatment plus liraglutide. Patients were randomized within 24 hours of TIA or AIS symptom onset and received the liraglutide within 1 hour after completing randomization. All the patients were given standard guideline-based treatment.

Follow-up period: NIHSS score was assessed at baseline, 7±1 days, and 90 ± 7 days after randomization. The mRS score was assessed at baseline, 30 ± 3 days, and 90 ± 7 after randomization. All concomitant medications, adverse events, stroke recurrence, and other vascular events of each visit were recorded since the last visit. All the adverse events of included subjects should be recorded and tracked until properly resolved. All the serious adverse events of included subjects should be recorded and tracked,

even if the subjects have finished the trial, until the events were resolved, or stabilization judged by the investigator.
All the adverse events of included subjects should be recorded and tracked until properly resolved.
All the serious adverse events of included subjects should be recorded and tracked, even if the subjects have finished the trial, until the events were resolved, or stabilization judged by the investigator.

8. Description of safety assessment parameters

8.1 Evaluate clinical safety

Physical examinations, including neurological assessments, and laboratory tests were performed at baseline, 7±1 days, 30 ± 3 days, and 90 ± 7 days after randomization; the adverse event of each visit was collected after baseline visit.

Adverse events monitoring

All information regarding adverse events, whether reported by subjects, identified by investigators, or detected through physical examination, laboratory tests, and other methods, must be documented on the adverse events page of the case report form and managed in compliance with relevant regulations and reporting requirements.

Adverse Event (AE)

The adverse event refers to any negative medical occurrence experienced by a study participant during the study, which may not necessarily be linked to the treatment being administered. This encompasses new events or those that have worsened in terms of severity and frequency compared to the baseline, including abnormal findings from diagnostic tests such as laboratory examinations.

Note: Adverse event collection begins with signed informed consent.

Serious Adverse Event (SAE)

A serious adverse event is defined as any unfavorable medical occurrence, regardless of dosage, that satisfies one or more of the specified criteria:

Cause death (note: death is a consequence, not an event);

Life-threatening (Note: "Life-threatening" means that the subject is in immediate danger of death at the time of the event, not the assumption that death would have occurred if the event is more severe);

Cause significant or permanent disability or impairment of organ function;

Significant medical event or need for intervention;

Causing or prolonging hospitalization.

Note: Any event requiring hospitalization (or prolonged hospitalization) that occurs during the subject's participation in the study must be reported as a serious adverse event. The following circumstances leading to hospitalization are not considered serious adverse events:

Hospitalization for social reasons other than adverse events

Hospitalization for a scheduled surgery or other treatment or examination prior to study entry (must be recorded on the case report form)

8.2 Safety assessment

The safety evaluation of adverse events in clinical studies necessitates the assessment of the following factors:

Severity criteria for SAE;

The causal relationship between the event and the investigational drug;

Severity of incident;

Anticipation of events;

Causal relationship between events and the study drugs.

Regardless of serious adverse events or non-serious adverse events, the investigator must evaluate the relevance of the event to use the study drugs according to the following criteria: Evaluating the association between adverse events and study treatment.

5-level classification	Judgment criteria

Definitely related	The time of initiation of use is reasonably related to the time of occurrence of the adverse event; the adverse event corresponds to the known adverse reaction type of the study treatment; the occurrence of the adverse event cannot be explained by factors other than the study treatment (such as concomitant medication); the adverse events reduce or disappear after study treatment dose reduction or discontinuation; similar adverse events (non-essential) can occur with re-use of study treatment.
Probably related	The time of initiation of use is reasonably related to the time of occurrence of the adverse event; the adverse event corresponds to the known adverse reaction type of the study treatment; the occurrence of the adverse event can or cannot be explained by factors other than the study treatment (such as concomitant medication); the adverse event can or cannot reduce or disappear after study treatment dose reduction or discontinuation; it is not sure whether similar adverse events (non-essential) can occur with re-use of study treatment.
Probably unrelated	The time of initiation of use is not reasonably related to the time of occurrence of the adverse event; the adverse event corresponds to the known adverse reaction type of the study treatment; the occurrence of the adverse event can or cannot be explained by factors other than the study treatment (such as concomitant medication); the adverse event can or cannot reduce or disappear after study treatment dose reduction or discontinuation; it is not sure whether similar adverse events (non-essential) can occur with re-use of study treatment.
Definitely unrelated	The time of initiation of use is not reasonably related to the time of occurrence of the adverse event; the adverse event corresponds to the known adverse reaction type of the study treatment; the occurrence of the adverse event can or cannot be explained by factors other than the study treatment (such as concomitant medication); the adverse event can or cannot reduce or disappear after study treatment dose reduction or discontinuation; similar adverse events (non-essential) cannot occur with re-use of study treatment.
Unjudged	The judgment cannot be made due to incomplete, contradictory information or the fact that the information cannot be supplemented and verified.

Table of Correlation Evaluation Criteria for Adverse Events and Treatment Use

	Definitely related	Probably related	Probably unrelated	Definitely unrelated	Unjudged
Have a reasonable chronological order with the study drugs	+	+	+	-	?
For the known reaction type of the study drugs	+	+	-	-	?
Explanation for reasons other than available research equipment	-	±	±	+	?
Responses lessen after decompression of study drugs	+	±	±	-	?

The reaction reappears after the study drugs is reused	+ or?	?	?	-	?

Note: "+" in the table is affirmative; "-" is negative; "±" is difficult to affirm or deny; "?" indicates that the situation is unknown.

Severity Criteria

Severity should be assessed according to the following graded descriptions:

Mild: No symptoms or discomfort; does not interfere with daily activities and function; usually does not require medication to relieve symptoms.

Moderate: Symptoms cause significant discomfort; daily activities and function are affected; study participation can be continued; intervention required to relieve symptoms.

Severe: Severe causes severe discomfort; symptoms result in loss of function and significantly interfere with daily activities; in severe cases, study treatment can be discontinued; symptomatic treatment and/or hospitalization are required.

The investigator should use clinical judgment to assess the severity of the event (e.g., abnormal laboratory results) from the subject's direct experience.

8.3 Safety report

All adverse events

All adverse events (AEs), regardless of their severity, nature, or causal relationship to the study treatment, should be documented in the original medical record and recorded on the clinical research form (CRF) from the time of signed informed consent until the last follow-up visit (90 days after initial drug use). When symptoms and signs are attributed to common causes, a diagnosis of the corresponding disease should be provided whenever possible (e.g., cough, runny nose, sneezing, sore throat, and headache should be reported as "upper respiratory tract infection"). The investigator is responsible for assessing the causal relationship between AEs and study treatment and documenting it on the CRF. All treatments administered for AEs must also be documented in accordance with PI requirements.

Any serious adverse events (SAEs) occurring during clinical research must be promptly reported within 24 hours to various entities including: ethics committee of the center; principal investigator of the center; sponsor; contract research organization; research team leader unit; and adverse reaction monitoring center. Additionally, reports must also be submitted within 7 calendar days to both the ethics committee and data safety monitoring committee of the team leader unit upon receiving notification. Investigators are required to complete an SAE form that includes information such as timing, severity, duration, actions taken, and outcomes associated with SAEs. Follow-up information regarding unresolved SAEs at study termination or subject early withdrawal should also be provided in writing within 24 hours following established procedures.

All SAE that did not resolve at study termination or subject early withdrawal must be followed up to any of the following:

1. Incident mitigation;
2. Events are stable;
3. If the baseline value is known, the event returns to the baseline value;
4. Events can be attributed to other drugs or factors not related to study;
5. It is unlikely that any further information will be available (subject or physician refuses to provide further information and remains lost to follow-up after various follow-up attempts).

Death

Death is the result of an event. The death of a subject in a clinical trial, regardless of whether the event was expected or drug-related, would be considered a serious adverse event. Events leading to death should be recorded in medical terms and reported on the eCRF. All causes of death (death diagnoses) must be reported as serious adverse events. Investigators should make every effort to obtain and send death certificates and autopsy reports to designated personnel.

Pregnancy

If the researcher finds that the subject is pregnant during the research, the researcher should report it to the clinical research supervisor and notify the project leader by phone. Abnormal pregnancy results, considered serious adverse events, should be reported in accordance with the

Serious Adverse Event Reporting Procedure.

Abnormal laboratory test results

During the study, laboratory test results meeting the following conditions must be considered as adverse events (AEs) and documented using medical terminology in the AE section of the Case Report Form (CRF).

1. Accompanying clinical symptoms;
2. Causing a change in the trial protocol (e.g., treatment interruption or discontinuation);
3. lead to a change in medical intervention or concomitant treatment;
4. Clinically significant as judged by the investigator (medical and scientific methods should be used to judge whether an isolated laboratory abnormality is an AE).

When the AE meets the serious criteria, it should be reported in accordance with the SAE reporting procedure.

9. Statistics

9.1 Sample size

Based on the other trial, the 90-day risk of the stroke recurrence risk is about 12.8% among high-risk TIA or minor stroke patients with T2DM treated within 24 hours of symptom onset [15]. The sample size formula was based on the comparisons of proportions of both groups. We hypothesize that the risk of stroke recurrence was reduced by 33.3% in the liraglutide group. A sample size of 1708 patients will have 80% power to detect a two-sided α-error of 0.05 and 5% patient loss or medication non-adherence.

9.2 Statistical analysis plan

Data analyses were carried out in the intention-to-treat population, defined as all randomized patients. Baseline data will refer to measurements taken prior to drug administration, with descriptive statistics as appropriate. Independent sample t-tests will be used to compare continuous variables with a normal distribution between groups, while the Mann–Whitney U test will be applied to non-normally distributed variables. Categorical variables will be compared using either the Chi-squared test or Fisher’s exact test, with counts and percentages used for descriptive statistics.

Kaplan-Meier estimates will be used to assess the cumulative stroke risk (ischemic or hemorrhagic) during the 90-day follow-up. Hazard ratios (HRs) with 95% confidence intervals (CIs) will be estimated using Cox proportional hazards models to evaluate treatment effects, while the log-rank test will be employed to compare survival curves between groups. Trial centers will be included as a random effect in the model. Centers with less than 10 subjects will be pooled with larger centers within the same geographic region so that centers are of a reasonable size for the purpose of the statistical analyses. For patients experiencing multiple events of the same type, only the time to the first event will be considered. Patients who do not experience the primary outcome will be censored at the earliest of the following time points: death, last known contact, or 90 days. All statistical analyses will be performed using two-tailed tests, with the P value of <0.05 considered statistically significant. Statistical analysis will be performed with SAS software version 9.4 (SAS Institute).

Stratification: The primary endpoint in the LAMP study will further be stratified by age (<65 years vs. ≥65 years), sex (male vs. female), BMI (<25 vs. ≥25), Qualifying event (stroke vs. TIA), previous hypertension (Yes vs. NO), Previous ischemic stroke or TlA (Yes vs. NO), Current smoker (Yes vs. No). Differences of primary endpoint in above specific stratifications will be assessed by testing for interaction of the pre-set baseline variable with primary endpoint.

9.3 Analysis population

Full Analysis Set (FAS)

According to the basic principle of intention-to-treat (ITT), all patients who have been enrolled, randomized and had the record of at least one-day treatment of study drugs will be included.

Subjects missing outcome data will be censored at the last follow-up assessment time (end of study or last visit preceding loss to follow up). This population will be the primary population for analyses of efficacy.

Per Protocol Set (PPS)

Per Protocol Set (PPS) is a subset of FAS. All patients with finishing the treatment or without violating the trail program seriously are included in PPS. The exact definition of a serious violation will be finalized at the time of data review and may generally include (but is not limited to) the

following criteria: non-compliance with the primary inclusion criteria, concomitant interference treatment after enrollment, poor compliance, and exceed the time window of follow-up seriously and so on. A partial protocol violator will be included in the Per Protocol Population up to the time of their violation. For the Per Protocol Population, participants will be analyzed according to the treatment received, providing the same treatment was taken for the duration of the study. If study medication was changed, then the participant will be considered a partial protocol violator (from the point of change onwards).

Safety Set (SS)

All patients who received at least 1-time of study drug according to the study protocol and safety assessment available will be included in the safety population. This population will be used for safety analysis.

10. Study organization

The executive committee was responsible for the design, interpretation, oversight, and management of the trial's conduct and analysis, including the development of the protocol and protocol amendments. The Clinical event committee conduct regular evaluations of clinical endpoint. An independent data and safety monitoring committee supervised the progression and conduct of the trial, ensuring the overall integrity of the trial. Statistical analysis was performed by the statistical and data management association.

11. Data management and monitoring

11.1 Training of study site personnel

The principle investigator will arrange a training session at each site prior to the enrollment of the first patients. The training will encompass all study-related personnel, including medical, nursing, and other staff members. The topics covered will include instructions on the trial protocol, investigator's brochure, scale evaluation, utilization of electronic data capture system, procedure for reporting serious adverse events (SAEs), among others.

11.2 Monitoring of the study

The principle investigator's representative will maintain regular communication with the study sites and be readily available to provide information about the study as needed by the investigator. The routine visits will encompass the following tasks: Ensuring adherence to the protocol and accurate documentation of data in the Case Report Forms (CRFs). The independent data and safety monitoring committee verify data validity, including timely receipt of informed consent, clinical data, laboratory results, neuroimaging data, and evaluate neurological function (NIHSS, mRS) at baseline and follow-up.

Data collection was the responsibility of the investigators, while site monitoring and data collation were undertaken by the clinical coordinating center. An independent data and safety monitoring committee supervised the progression and conduct of the trial, ensuring the overall integrity of the trial.

Appendix

Appendix 1: Definitions of stroke events and vascular events

Stroke	Acute symptoms and signs of neurological defect caused by sudden abnormality of the blood supply. Damage of focal or whole brain, spinal or retinal vascular damage, which is related to cerebral circulation disorder.
Ischemic stroke	Definitions: (1) Symptoms or imaging evidence of acute newly onset focal neurologic deficit last for more than 24 hours after excluding other non-ischemic reasons, such as brain infection, head trauma, brain tumor, epilepsy, severe metabolic diseases, degeneration diseases or adverse effect of medications; or (2) Acute brain or retinal ischemic event with focal symptoms or signs lasts for less than 24 hours after excluding other causes with imaging evidence of new infarction; or (3) Progression of original vascular ischemic stroke (NIHSS increased ≥ 4 from baseline score after excluding hemorrhagic transformation or symptomatic intracerebral hemorrhage after cerebral infarction) lasts over 24 hours with new ischemic lesion on brain MRI or CT. Which would be classified by ASCO etiology standard.
Transient ischemic attack	A brief episode of neurological dysfunction caused by focal brain or retinal ischemia, with clinical symptoms typically lasting less than 24 hours, and without evidence of acute infarction, after excluding other non-ischemic reasons, such as brain infection, head trauma, brain tumor, epilepsy, severe metabolic diseases, degeneration diseases or adverse effect of medications.
Hemorrhagic stroke	Hemorrhagic stroke was defined as focal or whole brain or spine damage caused by non-traumatic bleeding into the brain parenchyma, intraventricular or subarachnoid.
Myocardial infarction	Third universal definition of myocardial infarction（Thygesen 2012）<br>The term acute myocardial infarction (MI) should be used when there is evidence of myocardial necrosis in a clinical setting consistent with acute myocardial ischemia. Under these conditions any one of the following

	criteria meets the diagnosis for MI: 1、Detection of a rise and/or fall of cardiac biomarker values [preferably cardiac troponin (cTn)] with at least one value above the 99th percentile upper reference limit (URL) and with at least one of the following: (1) Symptoms of ischemia. (2) New or presumed new significant ST-segment–T wave (ST–T) changes or new left bundle branch block (LBBB). (3) Development of pathological Q waves in the ECG. (4) Imaging evidence of new loss of viable myocardium or new regional wall motion abnormality (5) Identification of an intracoronary thrombus by angiography or autopsy. 2、Cardiac death with symptoms suggestive of myocardial ischemia and presumed new ischemic ECG changes or new LBBB, but death occurred before cardiac biomarkers were obtained, or before cardiac biomarker values would be increased. 3、Percutaneous coronary intervention (PCI) related MI is arbitrarily defined by elevation of cTn values (>5×99th percentile URL) in patients with normal baseline values (≤99th percentile URL) or a rise of cTn values >20% if the baseline values are elevated and are stable or falling. In addition, either (1) symptoms suggestive of myocardial ischemia or (2) new ischemic ECG changes or (3) angiographic findings consistent with a procedural complication or (4) imaging demonstration of new loss of viable myocardium or new regional wall motion abnormality are required. 4、Stent thrombosis associated with MI when detected by coronary angiography or autopsy in the setting of myocardial ischemia and with a rise and/or fall of cardiac biomarker values with at least one value above the 99th percentile URL. 5、Coronary artery bypass grafting (CABG) related MI is arbitrarily defined by elevation of cardiac biomarker values (>10×99th percentile URL) in patients with normal baseline cTn values (≤99th percentile URL). In addition, either (1) new pathological Q waves or new LBBB, or (2) angiographic documented new graft or new native

	coronary artery occlusion, or (3) imaging evidence of new loss of viable myocardium or new regional wall motion abnormality.
Vascular death	Vascular death include death due to stroke, cardiac sudden death, death caused by acute myocardial infarction, death caused by heart failure, death caused by pulmonary embolism, death caused by cardial/cerebral interventions or operations (not caused by myocardial infarction) and death caused by other cardiovascular Vascular death diseases. (Arrhythmia irrelevant to cardiac sudden death, rupture of aortic aneurysm or peripheral artery disease). Unexplained death happened within 30 days after stroke, myocardial infarction or cardiovascular/cerebral vascular operation will be considered as stroke, myocardial infarction and accidental death caused by operation separately.

Appendix 2: Criteria for the diagnosis of diabetes[16]

FPG ≥126 mg/dL (7.0 mmol/L). Fasting is defined as no caloric intake for at least 8 h.*

OR

2-h PG ≥200 mg/dL (11.1 mmol/L) during OGTT. The test should be performed as described by the WHO, using a glucose load containing the equivalent of 75-g anhydrous glucose dissolved in water. *

OR

A1C ≥6.5% (48 mmol/mol). The test should be performed in a laboratory using a method that is NGSP certified and standardized to the DCCT assay. *

OR

In a patient with classic symptoms of hyperglycemia or hyperglycemic crisis, a random plasma glucose ≥200 mg/dL (11.1 mmol/L).

*In the absence of unequivocal hyperglycemia, diagnosis requires two abnormal test results from the same sample or in two separate test samples.

Appendix 3: National Institutes of Health Stroke Scale (NIHSS)[17]

**Instructions**	**Scale Definition**	**Score**
**1a. Level of Consciousness:** The investigator must choose a response if a full evaluation is prevented by such obstacles as an endotracheal tube, language barrier, orotracheal trauma/bandages. A 3 is scored only if the patient makes no movement (other than reflexive posturing) in response to noxious stimulation.	0 Alert; keenly responsive.<br>1 Not alert; but arousable by minor stimulation to obey, answer, or respond.<br>2 Not alert; requires repeated stimulation to attend or is obtunded and requires strong or painful stimulation to make movements (not stereotyped).<br>3 Responds only with reflex motor or autonomic effects or totally unresponsive, flaccid, and flexic.	____
**1b. LOC Questions:** The patient is asked the month and his/her age. The answer must be correct - there is no partial credit for being close. Aphasic and stuporous patients who do not comprehend the questions will score 2. Patients unable to speak because of endotracheal intubation, orotracheal trauma, severe dysarthria from any cause, language barrier, or any other problem not secondary to aphasia are given a 1. It is important that only the initial answer be graded and that the examiner not "help" the patient with verbal or non-verbal cues.	0 Answers both questions correctly.<br>1 Answers one question correctly.<br>2 Answers neither question correctly.	____
**1c. LOC Commands:** The patient is asked to open and close the eyes and then to grip and release the non-paretic hand. Substitute another one step command if the hands cannot be used. Credit is given if an unequivocal attempt is made but not completed due to weakness. If the patient does not respond to command, the task should be demonstrated to him or her (pantomime), and the result scored (i.e., follows none, one or two commands). Patients with trauma, amputation, or other physical impediments should be given suitable one-step commands. Only the first attempt is scored.	0 Performs both tasks correctly.<br>1 Performs one task correctly.<br>2 Performs neither task correctly.	____
**2. Best Gaze:** Only horizontal eye movements will be tested. Voluntary or reflexive (oculocephalic) eye movements will be scored, but caloric testing is not done. If the patient has a conjugate deviation of the eyes that can be overcome by voluntary or reflexive activity, the score will be 1. If a patient has an isolated peripheral nerve paresis (CN III, IV or VI), score a 1. Gaze is testable in all aphasic patients. Patients with ocular trauma, bandages, pre-existing blindness, or other disorder of visual acuity or fields should be tested with reflexive movements, and a choice made by the investigator. Establishing eye contact and then moving about the patient from side to side will occasionally clarify the presence of a partial gaze palsy.	0 Normal.<br>1 Partial gaze palsy; gaze is abnormal in one or both eyes but forced deviation or total gaze paresis is not present.<br>2 Forced deviation, or total gaze paresis not overcome by the oculocephalic maneuver.	____<br>____

**3. Visual:** Visual fields (upper and lower quadrants) are tested by confrontation, using finger counting or visual threat, as appropriate. Patients may be encouraged, but if they look at the side of the moving fingers appropriately, this can be scored as normal. If there is unilateral blindness or enucleation, visual fields in the remaining eye are scored. Score 1 only if a clear-cut asymmetry, including quadrantanopia, is found. If patient is blind from any cause, score 3. Double simultaneous stimulation is performed at this point. If there is extinction, patient receives a 1, and the results are used to respond to item 11.	0 No visual loss.<br>1 Partial hemianopia.<br>2 Complete hemianopia.<br>3 Bilateral hemianopia (blind including cortical blindness).	____
**4. Facial Palsy**: Ask – or use pantomime to encourage – the patient to show teeth or raise eyebrows and close eyes. Score symmetry of grimace in response to noxious stimuli in the poorly responsive or non-comprehending patient. If facial trauma/bandages, orotracheal tube, tape or other physical barriers obscure the face, these should be removed to the extent possible.	0 Normal symmetrical movements.<br>1 Minor paralysis (flattened nasolabial fold, asymmetry on smiling).<br>2 Partial paralysis (total or near-total paralysis of lower face).<br>3 Complete paralysis of one or both sides (absence of facial movement in the upper and lower face).	____
**5. Motor Arm:** The limb is placed in the appropriate position: extend the arms (palms down) 90 degrees (if sitting) or 45 degrees (if supine). Drift is scored if the arm falls before 10 seconds. The aphasic patient is encouraged using urgency in the voice and pantomime, but not noxious stimulation. Each limb is tested in turn, beginning with the non-paretic arm. Only in the case of amputation or joint fusion at the shoulder, the examiner should record the score as untestable (UN), and clearly write the explanation for this choice.	0 No drift; limb holds 90 (or 45) degrees for full 10 seconds.<br>1 Drift; limb holds 90 (or 45) degrees, but drifts down before full 10 seconds; does not hit bed or other support.<br>2 Some effort against gravity; limb cannot get to or maintain (if cued) 90 (or 45) degrees, drifts down to bed, but has some effort against gravity.<br>3 No effort against gravity; limb falls. 4 No movement.<br>UN Amputation or joint fusion, explain:<br>______________________________	5a. Left Arm<br>____<br>5b. Right Arm<br>____
**6. Motor Leg:** The limb is placed in the appropriate position: hold the leg at 30 degrees (always tested supine). Drift is scored if the leg falls before 5 seconds. The aphasic patient is encouraged using urgency in the voice and pantomime, but not noxious stimulation. Each limb is tested in turn, beginning with the non-paretic leg. Only in the case of amputation or joint fusion at the hip, the examiner should record the score as untestable (UN), and clearly write the explanation for this choice.	0 No drift; leg holds 30-degree position for full 5 seconds.<br>1 Drift; leg falls by the end of the 5-second period but does not hit bed.<br>2 Some effort against gravity; leg falls to bed by 5 seconds but has some effort against gravity.<br>3 No effort against gravity; leg falls to bed immediately.<br>4 No movement.<br>UN Amputation or joint fusion, explain:<br>___________________________	6a. Left Leg<br>____<br>6b. Right Leg<br>____

**7. Limb Ataxia:** This item is aimed at finding evidence of a unilateral cerebellar lesion. Test with eyes open. In case of visual defect, ensure testing is done in intact visual field. The finger-nose-finger and heel-shin tests are performed on both sides, and ataxia is scored only if present out of proportion to weakness. Ataxia is absent in the patient who cannot understand or is paralyzed. Only in the case of amputation or joint fusion, the examiner should record the score as untestable (UN), and clearly write the explanation for this choice. In case of blindness, test by having the patient touch nose from extended arm position.	0 Absent.<br>1 Present in one limb.<br>2 Present in two limbs.<br><br>UN Amputation or joint fusion, explain:<br><br>___________________________	____
**8. Sensory:** Sensation or grimace to pinprick when tested, or withdrawal from noxious stimulus in the obtunded or aphasic patient. Only sensory loss attributed to stroke is scored as abnormal and the examiner should test as many body areas (arms [not hands], legs, trunk, face) as needed to accurately check for hemisensory loss. A score of 2, “severe or total sensory loss,” should only be given when a severe or total loss of sensation can be clearly demonstrated. Stuporous and aphasic patients will, therefore, probably score 1 or 0. The patient with brainstem stroke who has bilateral loss of sensation is scored 2. If the patient does not respond and is quadriplegic, score 2. Patients in a coma (item 1a=3) are automatically given a 2 on this item.	0 Normal; no sensory loss.<br>1 Mild-to-moderate sensory loss; patient feels pinprick is less sharp or is dull on the affected side; or there is a loss of superficial pain with pinprick, but patient is aware of being touched.<br>2 Severe to total sensory loss; patient is not aware of being touched in the face, arm, and leg.	____
**9. Best Language:** A great deal of information about comprehension will be obtained during the preceding sections of the examination. For this scale item, the patient is asked to describe what is happening in the attached picture, to name the items on the attached naming sheet and to read from the attached list of sentences. Comprehension is judged from responses here, as well as to all of the commands in the preceding general neurological exam. If visual loss interferes with the tests, ask the patient to identify objects placed in the hand, repeat, and produce speech. The intubated patient should be asked to write. The patient in a coma (item 1a=3) will automatically score 3 on this item. The examiner must choose a score for the patient with stupor or limited cooperation, but a score of 3 should be used only if the patient is mute and follows no one-step commands.	0 No aphasia; normal.<br>1 Mild-to-moderate aphasia; some obvious loss of fluency or facility of comprehension, without significant limitation on ideas expressed or form of expression. Reduction of speech and/or comprehension, however, makes conversation about provided materials difficult or impossible. For example, in conversation about provided materials, examiner can identify picture or naming card content from patient’s response.<br>2 Severe aphasias; all communication is through fragmentary expression; great need for inference, questioning, and guessing by the listener. Range of information that can be exchanged is limited; listener carries burden of communication. Examiner cannot identify materials provided from patient response.<br>3 Mute, global aphasia; no usable speech or auditory comprehension.	____

461

**10. Dysarthria:** If patient is thought to be normal, an adequate sample of speech must be obtained by asking patient to read or repeat words from the attached list. If the patient has severe aphasia, the clarity of articulation of spontaneous speech can be rated. Only if the patient is intubated or has other physical barriers to producing speech, the examiner should record the score as untestable (UN), and clearly write an explanation for this choice. Do not tell the patient why he or she is being tested.	0 Normal.<br>1 Mild-to-moderate dysarthria; patient slurs at least some words and, at worst, can be understood with some difficulty.<br>2 Severe dysarthria; patient's speech is so slurred as to be unintelligible in the absence of or out of proportion to any dysphasia or is mute/anarthric.<br><br>UN Intubated or another physical barrier, explain:<br>______________________________	____
**11. Extinction and Inattention (formerly Neglect):** Sufficient information to identify neglect may be obtained during the prior testing. If the patient has a severe visual loss preventing visual double simultaneous stimulation, and the cutaneous stimuli are normal, the score is normal. If the patient has aphasia but does appear to attend to both sides, the score is normal. The presence of visual spatial neglect or anosagnosia may also be taken as evidence of abnormality. Since the abnormality is scored only if present, the item is never untestable.	0 No abnormality.<br>1 Visual, tactile, auditory, spatial, or personal inattention or extinction to bilateral simultaneous stimulation in one of the sensory modalities.<br>2 Profound hemi-inattention or extinction to more than one modality; does not recognize own hand or orients to only one side of space.	____
	**Total NIHSS:**	____

How to calculate the total NIHSS score?

When calculating the total score, the following should not be counted in the total score:

Items 5 and 6 - "9 Amputation or Joint Fusion" in Limb Movement

Item 7 - Item in Ataxia that identifies the location of the ataxia, i.e. "Left upper limb 1=Yes, 2=No, 9=Amputation or joint fusion, explain:" 。

Note:

Score according to the table and record the results. Do not change the score. The score reflects the actual situation of the patient, not what the doctor thinks the patient should be. Quickly check while recording results. Do not train the patient (e.g., repeatedly ask the patient to make an effort) unless instructed to do so. If some items are not evaluated, they should be explained in detail in the form. Unassessed items should be reviewed with surveillance video and discussed with the examiner

Attached picture

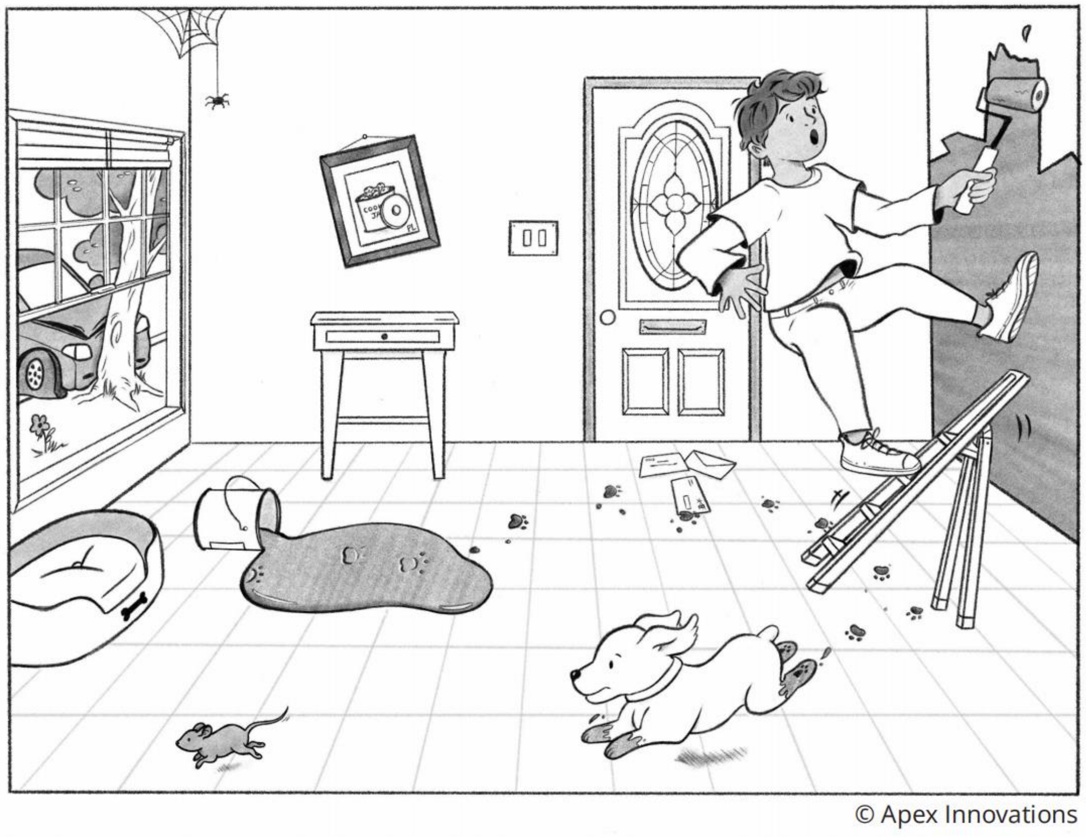

The naming sheet:
MAMA
TIP-TOP
FIFTY-FIFTY
THANKS
HUCKLEBERRY
BASEBALL PLAYER
CATERPILLAR

The list of sentences:
You know how.
Down to earth.
I got home from work.
Near the table in the dining room.
They heard him speak on the radio last night.

Appendix 4: Modified Rankin Score (mRS)

Grade	Description
0	No symptoms
1	Symptoms without any incapacity (able to perform all usual activities)
2	Mild incapacity (unable to perform all usual activities but able to look after his/her affairs alone)
3	Moderate incapacity (requires assistance but walks alone)
4	Severe incapacity (requires assistance for walking and physical body needs)
5	Severe incapacity (bedbound, incontinent, permanent surveillance required)
6	Death

Appendix 5: Montreal Cognitive Assessment (MoCA)[18]

MONTREAL COGNITIVE ASSESSMENT (MOCA®)
Version 8.1 English

Name:
Education: Date of birth:
Sex: DATE:

VISUOSPATIAL / EXECUTIVE | POINTS

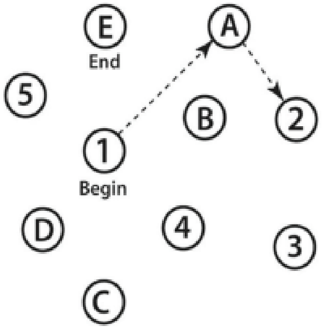

[ ]

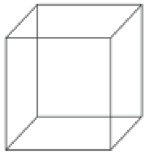

Copy cube [ ]

Draw CLOCK (Ten past eleven) (3 points)

[ ] Contour [ ] Numbers [ ] Hands

__/5

NAMING

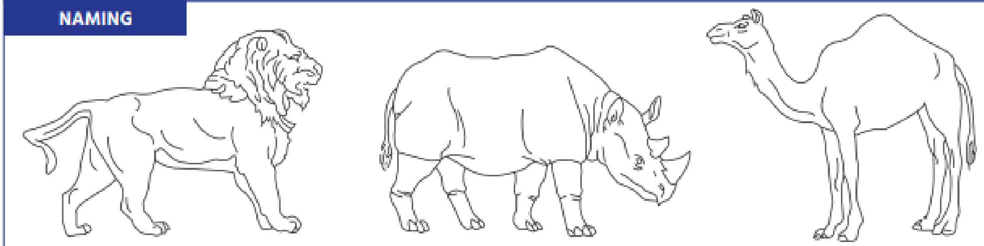

[ ] [ ] [ ]

__/3

MEMORY

Read list of words, subject must repeat them. Do 2 trials, even if 1st trial is successful. Do a recall after 5 minutes.

	FACE	VELVET	CHURCH	DAISY	RED
1ST TRIAL					
2ND TRIAL					

NO POINTS

ATTENTION

Read list of digits (1 digit/ sec.). Subject has to repeat them in the forward order. [ ] 2 1 8 5 4
Subject has to repeat them in the backward order. [ ] 7 4 2

__/2

Read list of letters. The subject must tap with his hand at each letter A. No points if ≥ 2 errors
[ ] F B A C M N A A J K L B A F A K D E A A A J A M O F A A B

__/1

Serial 7 subtraction starting at 100. [ ] 93 [ ] 86 [ ] 79 [ ] 72 [ ] 65
4 or 5 correct subtractions: **3 pts**, 2 or 3 correct: **2 pts**, 1 correct: **1 pt**, 0 correct: **0**

__/3

LANGUAGE

Repeat: I only know that John is the one to help today. [ ]
The cat always hid under the couch when dogs were in the room. [ ]

__/2

Fluency: Name maximum number of words in one minute that begin with the letter F. [ ] _____ (N ≥ 11 words)

__/1

ABSTRACTION

Similarity between e.g. banana - orange = fruit [ ] train - bicycle [ ] watch - ruler

__/2

DELAYED RECALL

(MIS)	Has to recall words WITH NO CUE	FACE	VELVET	CHURCH	DAISY	RED	Points for UNCUED recall only
X3		[ ]	[ ]	[ ]	[ ]	[ ]	
X2	Category cue						MIS = ____/15
X1	Multiple choice cue						

Memory Index Score (MIS)

__/5

ORIENTATION

[ ] Date [ ] Month [ ] Year [ ] Day [ ] Place [ ] City

__/6

© Z. Nasreddine MD www.mocatest.org

MIS: /15
(Normal ≥ 26/30)
Add 1 point if ≤ 12 yr edu

TOTAL __/30

Administered by: ______________________

Training and Certification are required to ensure accuracy

Reference

1. Wang Y, Liu M, Pu C. 2014 Chinese guidelines for secondary prevention of ischemic stroke and transient ischemic attack. *Int J Stroke*. Apr 2017;12(3):302-320. doi:10.1177/1747493017694391
2. Society CD. Chinese guideline for the prevention and treatment of type 2 diabetes mellitus (2017 edition). *Chin J Diabetes Mellitus*. 2018;10(1):4-67.
3. Jia Q, Zhao X, Wang C, et al. Diabetes and poor outcomes within 6 months after acute ischemic stroke: the China National Stroke Registry. *Stroke*. Oct 2011;42(10):2758-62. doi:10.1161/strokeaha.111.621649
4. Peters SA, Huxley RR, Woodward M. Diabetes as a risk factor for stroke in women compared with men: a systematic review and meta-analysis of 64 cohorts, including 775,385 individuals and 12,539 strokes. *Lancet*. Jun 7 2014;383(9933):1973-80. doi:10.1016/s0140-6736(14)60040-4
5. Lau LH, Lew J, Borschmann K, Thijs V, Ekinci EI. Prevalence of diabetes and its effects on stroke outcomes: A meta-analysis and literature review. *J Diabetes Investig*. May 2019;10(3):780-792. doi:10.1111/jdi.12932
6. Amarenco P, Lavallée PC, Labreuche J, et al. One-Year Risk of Stroke after Transient Ischemic Attack or Minor Stroke. *N Engl J Med*. Apr 21 2016;374(16):1533-42. doi:10.1056/NEJMoa1412981
7. Johnston SC, Gress DR, Browner WS, Sidney S. Short-term prognosis after emergency department diagnosis of TIA. *Jama*. Dec 13 2000;284(22):2901-6. doi:10.1001/jama.284.22.2901
8. Arsava EM, Kim GM, Oliveira-Filho J, et al. Prediction of Early Recurrence After Acute Ischemic Stroke. *JAMA Neurol*. Apr 2016;73(4):396-401. doi:10.1001/jamaneurol.2015.4949
9. Pan Y, Jing J, Li H, Wang Y, Wang Y, He Y. Abnormal glucose regulation increases stroke risk in minor ischemic stroke or TIA. *Neurology*. Oct 11 2016;87(15):1551-1556. doi:10.1212/wnl.0000000000003200
10. Jia Q, Zheng H, Zhao X, et al. Abnormal glucose regulation in patients with acute stroke across China: prevalence and baseline patient characteristics. *Stroke*. Mar 2012;43(3):650-7. doi:10.1161/strokeaha.111.633784
11. Marso SP, Daniels GH, Brown-Frandsen K, et al. Liraglutide and Cardiovascular Outcomes in Type 2 Diabetes. *N Engl J Med*. Jul 28 2016;375(4):311-22. doi:10.1056/NEJMoa1603827
12. Marso SP, Bain SC, Consoli A, et al. Semaglutide and Cardiovascular Outcomes in Patients with Type 2 Diabetes. *N Engl J Med*. Nov 10 2016;375(19):1834-1844. doi:10.1056/NEJMoa1607141
13. Powers WJ, Rabinstein AA, Ackerson T, et al. 2018 Guidelines for the Early Management of Patients With Acute Ischemic Stroke: A Guideline for Healthcare Professionals From the American Heart Association/American Stroke Association. *Stroke*. Mar 2018;49(3):e46-e110. doi:10.1161/str.0000000000000158
14. Johnston SC, Easton JD, Farrant M, et al. Platelet-oriented inhibition in new TIA and minor ischemic stroke (POINT) trial: rationale and design. *Int J Stroke*. Aug 2013;8(6):479-83. doi:10.1111/ijs.12129

15. Wang Y, Wang Y, Zhao X, et al. Clopidogrel with aspirin in acute minor stroke or transient ischemic attack. *N Engl J Med*. Jul 4 2013;369(1):11-9. doi:10.1056/NEJMoa1215340
16. American Diabetes A. 2. Classification and Diagnosis of Diabetes: Standards of Medical Care in Diabetes-2019. *Diabetes Care*. Jan 2019;42(Suppl 1):S13-S28. doi:10.2337/dc19-S002
17. Health NIo. NIH Stroke Scale (NIHSS). Accessed September 19, 2024, https://www.stroke.nih.gov/resources/nihss.pdf
18. Organization MT. Montreal Cognitive Assessment (MoCA). Accessed September 20, 2024, https://www.mocatest.org/
