# Supplementary material for: Liraglutide in Acute Minor Ischemic Stroke or High-Risk Transient Ischemic Attack With Type 2 Diabetes: The LAMP Randomized Clinical Trial
Source: JAMA Intern Med. 2025 Nov 3;186(1):46–54. doi: 10.1001/jamainternmed.2025.5684 (PMC12584062; doi:10.1001/jamainternmed.2025.5684)
Supplement: Supplement 2. — Statistical analysis plan [file jamainternmed-e255684-s002.pdf]

Version 1.0 of 2019

Liraglutide in Acute Minor Ischemic Stroke or High-risk Transient Ischemic Attack Patients with Type 2 Diabetes Mellitus (LAMP): A Multicenter, Controlled, Prospective, Randomized, Open-label, Blinded Endpoint (PROBE) Trial

Principle Investigator:

Anding Xu, Huili Zhu

Department of Neurology

the First Affiliated Hospital of Jinan University

Guangzhou, 510000, China

Tel: +8613392692160, +8613922165588

E-mail: tlil@jnu.edu.cn, zhlffff@163.com

Table of Contents

1. Introduction 3
2. Study Objective 3
3. Study Endpoint(s) 3
Primary Efficacy Endpoints: 3
Secondary Efficacy Endpoint: 3
Primary Safety Endpoint 3
Secondary Safety Endpoint 3
4. Statistical Hypotheses 4
5. Design 4
Planned Analyses 5
6. Sample size estimates 5
7. Analysis populations 6
Full Analysis Set (FAS) 6
Per Protocol Set (PPS) 6
Safety Set (SS) 7
8. Treatment comparisons 7
9. General considerations for data analyses 7
Multicenter Studies 7
Examination of Subgroups 8
Multiple Comparisons and Multiplicity 8
10. Data handling conventions 8
Premature Withdrawal and Missing Data 8
Event Rates 8
Time to Event Analysis 8
11. Study Population 9
Disposition of Subjects 9
Protocol Deviations 9
Demographic and Baseline Characteristics 9
12. Efficacy Analyses 11
Primary Efficacy Analysis 11
Main Model 11
Interactions with Subgroups 11
Secondary Efficacy Analyses 11
13. Safety Analyses 12
14. References 13

1. Introduction

This Statistical Analysis Plan (SAP) outlines the planned statistical analyses for the "Liraglutide in Acute Minor Ischemic Stroke or High-risk Transient Ischemic Attack Patients with Type 2 Diabetes Mellitus （LAMP）: A Multicenter, Controlled, Prospective, Randomized, Open-label, Blinded Endpoint (PROBE) Trial ".

This SAP is intended for the use of project team members and should be read in conjunction with the protocol.

2. Study Objective

The objective of the study is to investigate the safety and efficacy of the Glucagon-like peptide-1 (GLP-1) receptor agonists liraglutide in treating acute mild ischemic stroke (National Institutes of Health Stroke Scale [NIHSS] score ≤3) or high-risk transient ischemic attack (TIA) (ABCD2 score ≥4) patients with type 2 diabetes mellitus.

3. Study Endpoint(s)

Primary Efficacy Endpoints:

The proportion of patients who experience stroke recurrence (ischemic or hemorrhagic) within 90±7 days.

Secondary Efficacy Endpoint:

The percentage of patients experiencing new clinical vascular events at 90±7 days, including ischemic stroke, hemorrhagic stroke, TIA, myocardial infarction, and vascular death;

The proportion of patients achieving a modified Rankin Scale (mRS) score ≤1 or ≤2 points and evaluate changes in mRS scores at 90±7 days.

Primary Safety Endpoint

The rate of symptomatic intracerebral hemorrhage at 90±7 days.

Secondary Safety Endpoint

The proportion of pancreatitis at 90±7 days.

The percentage of hypoglycemic events (blood glucose <3.9 mmol/L) at 90±7 days.

The percentage of gastrointestinal disorders events at 90±7 days.

The percentage of pneumonia at 90±7 days.

90-day all-cause mortality

Adverse events, and severe adverse events through 90±7 days of follow-up.

4. Statistical Hypotheses

The primary endpoint for this study is new stroke event (ischemic stroke or hemorrhagic stroke) during the 90 days treatment period. The null hypothesis of no difference in this rate between the two treatment groups will be tested using a two-sided test at the 5% level of significance.

H0: $\lambda 1/\lambda 2=1$

H1: $\lambda 1/\lambda 2\neq 1$

Where λ1 is the rate of new stroke over the 90 days treatment period in the group treated with liraglutide and λ2 is the same endpoint in the group treated with standard treatment.

5. Design

The study is a Multicenter, Controlled, Prospective, Randomized, Open-label, Blinded Endpoint (PROBE) Trial to assess liraglutide's safety and effectiveness in reducing stroke recurrence and improving prognosis for acute mild ischemic stroke/ high-risk TIA patient with type 2 diabetes mellitus. Follow-up data were collected at 7 days, 30±3 days, and 90±7 days after randomization.

Eligibility criteria for the trial participants include patients aged 50 or older with type 2 diabetes mellitus, who have either an acute ischemic stroke with an NIHSS score ≤3 or a high-risk TIA with an ABCD2 score ≥4, and can receive the study drug within 24 hours of symptom onset or those experiencing their first stroke or with a history of previous stroke without any residual effects, ensuring that the current NIHSS score is unaffected and with an mRS score of ≤1. Signed informed consent is also required. Participants are excluded from the trial if they have any of the following conditions: 1) diagnosis of intracranial hemorrhagic diseases on baseline computed tomography 2) iatrogenic or cardiogenic stroke; 3) undergoing thrombolysis or endovascular treatment; 4) regular glucagon-like peptide-1 analogue use in last 90 days; 5) family/personal history of multiple endocrine neoplasia type 2 or familial medullary thyroid carcinoma; 6) current or past pancreatitis, inflammatory bowel disease, or a history of gastroparesis; 7) pregnant, lactating women, or patients likely or planning to become pregnant; 8) allergy to liraglutide or any of its excipients; 9) congestive heart failure (New York Heart Association class III-IV); 10) severe liver and kidney dysfunction (aspartate transaminase/alanine transaminase ratio and serum creatinine are 3 times higher than normal upper limit); 11) malignant tumors with an expected life expectancy of less than three months;

12) participation in other drug clinical trials within the past three months; or 13) any other condition that the investigator deems inappropriate for participation in this clinical study. All eligible patients will be randomized to receive either the liraglutide plus standard treatment group or the standard treatment group.

In the treatment(liraglutide) arm, the initial dosage of liraglutide was 0.6 mg per day, which was subsequently increased to 1.2 mg per day in the second week and further escalated to 1.8 mg per day in the third week through subcutaneous injection once daily. From then on, a consistent dosage of 1.8 mg per day was maintained until day 90. The control arm will not utilize liraglutide. Both arms received standard treatment according to the guideline.

The primary efficacy outcome is proportion of patients who experience stroke recurrence (ischemic or hemorrhagic) within 90±7 days.

Secondary efficacy outcomes included: 1)The percentage of patients experiencing new clinical vascular events at 90±7 days, including ischemic stroke, hemorrhagic stroke, TIA, myocardial infarction, and vascular death; 2) The proportion of patients achieving a modified Rankin Scale (mRS) score ≤1 or ≤2 points and evaluate changes in mRS scores at 90±7 days. The primary safety outcome was the rate of symptomatic intracerebral hemorrhage at 90±7 days.

The trial was approved by the ethics committee of the participating hospitals. The study was registered with ClinicalTrials.gov (NCT03948347).

Planned Analyses

The analyses that are detailed in this SAP will be performed only when the database has been locked, all protocol violators identified, and treatment allocations have been unblinded. Membership of the Full Analysis and Per Protocol populations will be determined using the rules set out in this SAP. At a date to be agreed within the project team, a data look will be performed. This will involve production of all data displays on a subset of the data using dummy treatment codes. These are produced purely as an aide to the pre-programming of the study and no unblinding will occur.

6. Sample size estimates

The study hypothesized that there is no difference in 90-day risk of stroke (ischemic or hemorrhagic) between the liraglutide plus standard treatment group or the standard treatment group.

The minimum necessary sample size in the trial is established by the requirement to detect the smallest expected, clinically meaningful treatment difference comparing the treatment with

placebo. Based on the other trial, the 90-day risk of the stroke recurrence risk is about 12.8% among high-risk TIA or minor stroke patients with T2DM treated within 24 hours of symptom onset [1]. The sample size formula was based on the comparisons of proportions of both groups. We hypothesize that the risk of stroke recurrence was reduced by 33.3% in the liraglutide group. A sample size of 1708 patients will have 80% power to detect a two-sided α-error of 0.05 and 5% patient loss or medication non-adherence.

7. Analysis populations

Full Analysis Set (FAS)

According to the basic principle of intention-to-treat (ITT), all patients who were originally randomly assigned, irrespective of whether they prematurely discontinued treatment or violated the protocol. Subjects missing outcome data will be censored at the last follow-up assessment time (end of study or last visit preceding loss to follow up). This population will be the primary population for analyses of efficacy.

Per Protocol Set (PPS)

Per Protocol Set (PPS) is a subset of FAS. All patients with finishing the treatment or without violating the trail program seriously are included in PPS. The exact definition of a serious violation will be finalized at the time of data review and may generally include (but is not limited to) the following criteria: non-compliance with the primary inclusion criteria, concomitant interference treatment after enrollment, poor compliance, and exceed the time window of follow-up seriously and so on. A partial protocol violator will be included in the Per Protocol Population up to the time of their violation. For the Per Protocol Population, participants will be analyzed according to the treatment received, providing the same treatment was taken for the duration of the study. If study medication was changed then the participant will be considered a partial protocol violator (from the point of change onwards).

Safety Set (SS)

All patients who received at least 1-time of study drug according to the study protocol and safety assessment available will be included in the safety population. This population will be used for safety analyses.

8. Treatment comparisons

The treatment comparison of interest in this study is between Liraglutide and blank control in acute ischemic stroke/TIA with Type 2 Diabetes.

9. General considerations for data analyses

All programming will be performed using SAS software version 9.4 (SAS Institute). All analysis output will use the following treatment group naming conventions and treatment order:

Liraglutide group： the initial dosage of liraglutide was 0.6 mg per day, which was subsequently increased to 1.2 mg per day in the second week and further escalated to 1.8 mg per day in the third week through subcutaneous injection once daily. From then on, a consistent dosage of 1.8 mg per day was maintained until day 90. If dose escalation results in unacceptable adverse effects, the intervals for dose escalation could be extended, treatment could be temporarily paused, or maintenance doses below the target dose of 1.8 mg per day could be considered.

Control group: liraglutide will not be used.

Both groups received standard treatment according to the guideline. Unless otherwise specified all significance tests will be 2-sided at the alpha=0.05 level and all confidence intervals will be 95%.

Multicenter Studies

In multicenter randomized controlled clinical study, there were some difference effect in different center due to different baseline, clinical practice or other factor, therefore, central effect analysis was required. Trial centers will be included as a random effect in the Cox proportional hazards model. Centers with less than 10 subjects will be pooled with larger centers within the same geographic region so that centers are of a reasonable size for the purpose of the statistical analyses.

Examination of Subgroups

The rate of new stroke (ischemic or hemorrhagic) at 90 days will be presented for each level of the covariates: age (<65 years vs. ≥65 years), sex (male vs. female), BMI (<25 vs. ≥25), Qualifying event (stroke vs. TIA), previous hypertension (Yes vs. NO), Previous ischemic stroke or TlA (Yes vs. NO), Current smoker (Yes vs. No). The extent to which the treatment effect varies across levels of each subgroup will be assessed through interaction tests.

Multiple Comparisons and Multiplicity

A single primary efficacy variable has been defined for this study, with all other efficacy variables identified as secondary or other. Similarly, only one treatment comparison is of interest in the study and therefore there are no requirements to adjust for multiple comparisons or multiple endpoints within this study.

10. Data handling conventions

Premature Withdrawal and Missing Data

If any subject withdraws prematurely from the study (prior to the final visit D90±7 days assessment), they are required to complete the withdrawal visit in the CRF. The reasons for withdrawal will be presented in a summary table. For the purposes of summaries and analysis of clinic visit data, this visit will be assigned to the next scheduled clinic visit for that subject, regardless of whether the date falls within the next visit window.

Subjects who withdraw before the end of the study, but who do provide at least one post-baseline measure for a particular endpoint, will be included in the analysis.

Subjects who do not attend any visits after randomization will be excluded from analysis of any endpoint, as no post-baseline data will be available.

Event Rates

The number of people and person-time of events should be recorded in detail and showing the event rate in 90 days of each treatment group in summary statement.

The event rate for each treatment group will be calculated as: the sum of number of event for all the patients / the sum of number of treatment periods for all the patients.

Time to Event Analysis

Differences between treatments in the risk of new stroke (ischemic or hemorrhagic) event and combined vascular events during maximum 90-day follow-up were assessed using standard

Kaplan-Meier time-to-event approaches. For patients experiencing multiple events of the same type, only the time to the first event will be considered. Patients who do not experience the primary outcome will be censored at the earliest of the following time points: death, last known contact, or 90 days.

11. Study Population

Disposition of Subjects

The number of subjects in each analysis population will be presented, subjects to be excluded from the Per Protocol population will be listed, and the total number of subjects attending each clinic visit will also be summarized by treatment group.

The number of subjects Randomized, completed and prematurely withdrawn from the study will be presented for each treatment group. The primary reasons for withdrawal both prior to and post randomization will also be presented.

A data display listing and summary of deviations from the inclusion/exclusion criteria will be presented for all subjects who were either entered or Randomized into the trial.

Protocol Deviations

Subject data will be examined for evidence of protocol violators in order to assess how well the protocol was followed. Inclusion and exclusion criteria are detailed in the study protocol. Subjects who commit protocol violations will be included in the ITT Population but excluded from the Per Protocol Population. These protocol violations will be shown in a listing.

Subjects can either be full or partial protocol violators. A full protocol violator is completely excluded from the Per Protocol Population. A partial protocol violator has only some data excluded. For subjects who violated the protocol during the treatment period due to unpermitted changes in the medication or prohibited concurrent medication, the analysis will only use data recorded prior to the violation.

For all violations which reference the treatment period, the treatment start date will be used as the reference date.

A listing of all possible protocol violators will be produced for clinical review. The final list of subjects who are protocol violators and are therefore excluded from the Per-Protocol population will be agreed by the study team prior to unblinding the study.

Demographic and Baseline Characteristics

The following information will be listed and summarized for subjects in each treatment group:

demographic characteristics, medical history, brain imaging, neurological measurements and haematological examination, as detailed in the study protocol.

The continuous data followed normal distribution will be presented as mean and standard deviation, and the continuous data followed skewness distribution will be presented as median and interquartile range; categorical data will be presented as n(%). T-test or Wilcoxon rank sum test will be used for comparison between two continuous data, and Chi-squared tests or Fisher exact test will be used for comparison between two categorical data.

12. Efficacy Analyses

Primary Efficacy Analysis

The primary endpoint is the rate of new stroke (both ischemic and hemorrhagic) reported during the 90-day treatment period. ITT will be the primary population for efficacy analyses.

Main Model

The time to first new stroke (both ischemic and hemorrhagic) reported during the 90-day treatment period will be summarized by treatment group using Kaplan-Meier estimates. The hazard ratio for the treatment comparison will be derived using a Cox's proportional hazards model, including the pooled study center as a random effect. The hazards ratios with 95% CI will be reported. This will also be presented graphically on a Kaplan-Meier curve.

Interactions with Subgroups

Summary tables will be produced for the predefined subgroups and interactions between treatment and these subgroups will be investigated, using a Cox proportional hazards model. A separate model will be used for each interaction to determine its significance. This will also be presented graphically on a forest plot.

The predefined subgroups including:

- Age (<65 years vs. ≥65years)
- Sex (Male vs. Female)
- BMI (<25, ≥25)
- Qualifying event
- Previous hypertension
- Previous ischemic stroke or TIA
- Current smoker

Secondary Efficacy Analyses

Rate of new clinical vascular events (ischemic stroke, hemorrhagic stroke, TIA, myocardial infarction, or vascular death) during the 90-day treatment period

The rate of new clinical vascular events occurring within 90 days will be analyzed using a Cox proportional hazard model, with the pooled study center as a random effect. A combined

vascular event is defined as any of the 5 following events: ischemic stroke, hemorrhagic stroke, TIA, myocardial infarction, or vascular death. This will also be presented graphically on a Kaplan-Meier curve. The hazard ratios with 95% CIs will be reported.

Excellent functional outcome (mRS ≤ 1) and favorable functional outcome (mRS ≤ 2) at 90 days

The rate of excellent functional outcome (mRS ≤ 1) and favorable functional outcome (mRS ≤ 2) will be compared using binary logistic analysis. The odds ratios with 95% CIs will be reported. The rate of mRS ≤ 1 and mRS ≤ 2 will be calculated for each treatment group.

13. Safety Analyses

All analyses of safety data will be carried out using the safety set (SS) population. Safety outcomes include symptomatic intracerebral hemorrhage (sICH) as defined by European Cooperative Acute Stroke Study II (ECASS II), hypoglycemic events, gastrointestinal disorders, pneumonia, pancreatitis within 90 days, and 90-day all-caused death. The comparison of safety outcomes between two groups will be analyzed using a Cox proportional hazard model similar to that in the previous section. The hazard ratios with 95% CIs will be reported.

14. References

1. Wang, Y., et al., *Clopidogrel with aspirin in acute minor stroke or transient ischemic attack.* N Engl J Med, 2013. **369**(1): p. 11-9.
