# Supplementary material for: Liraglutide in Acute Minor Ischemic Stroke or High-Risk Transient Ischemic Attack With Type 2 Diabetes: The LAMP Randomized Clinical Trial
Source: JAMA Intern Med. 2025 Nov 3;186(1):46–54. doi: 10.1001/jamainternmed.2025.5684 (PMC12584062; doi:10.1001/jamainternmed.2025.5684)
Supplement: Supplement 3. — eAppendix 1. Recruitment by Site in LAMP Trial eAppendix 2. Committee Members eMethods. eFigure 1. Trial Profile eFigure 2. Distribution of Modified Rankin Scale Scores at 90 Days in the Intention-to-Treat eFigure 3. Distribution of Modified Rankin Scale Scores at 90 Days in the Per-Protocol Analysis eFigure 4. Primary Outcome by Prespecified Subgroups in the Per-Protocol Analysis eTable 1. Criteria for the diagnosis of diabetes eTable 2. Definitions of stroke events and vascular events eTable 3. Baseline Characteristics of the Population in the Per-Protocol Analysis eTable 4. The patient's glycemic control and antidiabetic drug use status in Full Analysis Set eTable 5. Trial Outcomes in the Per-Protocol Analysis [file jamainternmed-e255684-s003.pdf]

Supplemental Online Content

Zhu H, Yang B, Lu L, et al; LAMP Investigators. Liraglutide in acute minor ischemic stroke or high-risk transient ischemic attack with type 2 diabetes: the LAMP randomized clinical trial. *JAMA Intern Med*. Published online November 3, 2025. doi:10.1001/jamainternmed.2025.5684

eAppendix 1. Recruitment by Site in LAMP Trial
eAppendix 2. Committee Members
eMethods.
eFigure 1. Trial Profile
eFigure 2. Distribution of Modified Rankin Scale Scores at 90 Days in the Intention-to-Treat
eFigure 3. Distribution of Modified Rankin Scale Scores at 90 Days in the Per-Protocol Analysis
eFigure 4. Primary Outcome by Prespecified Subgroups in the Per-Protocol Analysis
eTable 1. Criteria for the diagnosis of diabetes
eTable 2. Definitions of stroke events and vascular events
eTable 3. Baseline Characteristics of the Population in the Per-Protocol Analysis
eTable 4. The patient's glycemic control and antidiabetic drug use status in Full Analysis Set
eTable 5. Trial Outcomes in the Per-Protocol Analysis

This supplemental material has been provided by the authors to give readers additional information about their work.

© 2025 Zhu H et al. *JAMA Internal Medicine*.

eAppendix 1: Recruitment by Site in LAMP Trial

NO.	Inclusion site	Number of patients recruited
1	Department of Neurology, the First Affiliated Hospital of Jinzhou Medical University	88
2	Department of Neurology, People's Hospital of Longmen County	83
3	Department of Neurology, People's Hospital of Xinfeng County	63
4	Department of Neurology, the Second Affiliated Hospital of Harbin Medical University	49
5	Department of Neurology, the First Hospital of Putian	48
6	Department of Neurology, the First Affiliated Hospital of Jinan University	44
7	Department of Neurology, The Fourth Affiliated Hospital of Guangzhou Medical University, Guangzhou	40
8	Department of Neurology, Meizhou People's Hospital	36
9	Department of Neurology, Liuyang Jili Hospital	25
10	Department of Neurology, Affiliated Hospital of Youjiang Medical College for Nationalities	25
11	Department of Neurology, Guangzhou Red Cross Hospital	19

© 2025 Zhu H et al. *JAMA Internal Medicine*.

12	Department of Neurology, Guangdong Clifford Hospital Co., LTD	17
13	Department of Neurology, Zhongshan People's Hospital	15
14	Department of Neurology, the First People's Hospital of Zhaoqing	14
15	Department of Neurology, the First Affiliated Hospital of Gannan Medical College	10
16	Department of Neurology, Dongguan People's Hospital	10
17	Department of Neurology, Houjie Hospital, Dongguan City	9
18	Department of Neurology, Ganzhou People's Hospital	8
19	Department of Neurology, the Sixth Affiliated Hospital of Jinan University	7
20	Department of Neurology, Panyu Central Hospital, Guangzhou	6
21	Department of Neurology, He Xian Memorial Hospital, Panyu District, Guangzhou	5
22	Department of Neurology, Changde Hospital, Xiangya School of Medicine, Central South University（The first people's hospital of Changde city)	4
23	Department of Neurology, Shunde Hospital Affiliated to Jinan University	3
24	Department of Neurology, Jiangmen Central Hospital	3
25	Department of Neurology, Zhuhai People's Hospital	2

© 2025 Zhu H et al. *JAMA Internal Medicine*.

26	Department of Neurology, Sun Yat-sen Memorial Hospital, Sun Yat-sen University	2
27	Department of Neurology, Xiangya Hospital, Central South University	1

© 2025 Zhu H et al. *JAMA Internal Medicine*.

eAppendix 2: Committee Members

Steering Committee

1. Yong-Jun Wang (Chairman, Tiantan Hospital, Capital Medical University, Beijing, China)
2. An-Ding Xu (Department of Neurology, the First Affiliated Hospital of Jinan University, Guangzhou, China)
3. Hui-Li Zhu (Department of Neurology, the First Affiliated Hospital of Jinan University, Guangzhou, China)
4. Xiang-Bin Wang (Division of Endocrinology, Metabolism & Nutrition, Department of Medicine, Rutgers University-Robert Wood Johnson Medical School, New Brunswick, NJ, USA)
5. Hao Li (Tiantan Hospital, Capital Medical University, Beijing, China)
6. Li-An Huang (Department of Neurology, the First Affiliated Hospital of Jinan University, Guangzhou, China)
7. Yu-Sheng Zhang (Department of Neurology, the First Affiliated Hospital of Jinan University, Guangzhou, China)

Data Monitoring Committee

1. David-Wang (Chairman, St. Joseph's Hospital and Medical Center, Phoenix, USA)
2. An-Xin Wang (Medical Statistics, Tiantan Hospital, Capital Medical University, Beijing, China)
3. Jun Lv (Medical Statistics, Department of Clinical Research, The First Affiliated Hospital of Jinan University, Guangzhou, China)

Executive committee

1. An-Ding Xu (Chairman, Department of Neurology, the First Affiliated Hospital of Jinan University, Guangzhou, China)
2. Hui-Li Zhu (Co-chairman, Department of Neurology, the First Affiliated Hospital of Jinan University, Guangzhou, China)
3. Ru-Bo Sui (Local Principal Investigator, Department of Neurology, the First Affiliated Hospital of Jinzhou Medical University, Jinzhou, China)
4. Li-Hua Wang (Local Principal Investigator, Department of Neurology, The Second Affiliated Hospital of Harbin Medical University, Harbin, China)

© 2025 Zhu H et al. *JAMA Internal Medicine*.

5. Bei-Yu Zhang (Local Principal Investigator, Department of Neurology, Liuyang Jili Hospital, Liuyang, China)
6. Su-Ping Zhang (Local Principal Investigator, Department of Neurology, Guangzhou Red Cross Hospital, Guangzhou, China)
7. Wen-Jun Wu (Local Principal Investigator, Department of Neurology, Zhongshan People's Hospital, Zhongshan, China)
8. You-Jia Li (Local Principal Investigator, Department of Neurology, the First People's Hospital of Zhaoqing, Zhaoqing, China)
9. Ying Huang (Local Principal Investigator, Department of Neurology, the First Affiliated Hospital of Gannan Medical College, Ganzhou, China)
10. Xiao-Yun Huang (Local Principal Investigator, Department of Neurology, Houjie Hospital, Dongguan, Dongguan, China)
11. Wen-Yan Zhuo (Local Principal Investigator, Department of Neurology, Zhuhai People's Hospital, Zhuhai, China)
12. Yu-Qin Shen (Local Principal Investigator, Department of Neurology, Sun Yat-sen Memorial Hospital, Sun Yat-sen University, Guangzhou, China)
13. Jian-Xia (Local Principal Investigator, Department of Neurology, Xiangya Hospital, Central South University, Changsha, China)

Clinical Events Committee

1. Yan-Sheng Li (Chairman, Department of Neurology, Renji Hospital affiliated to Shanghai Jiaotong University School of Medicine, Shanghai, China)
2. Kang-Ning Chen (Department of Neurology, The First Affiliated Hospital of Army Medical University, Chongqing, China)
3. Bo Hu (Department of Neurology, Union Hospital Affiliated to Tongji Medical College, Huazhong University of Science and Technology, Wuhan, Chian)

Institution Human Research Ethics Committee

1. Zhen-Gang Cha (Chairman, the First Affiliated Hospital of Jinan University, Guangzhou, China)
2. Liang-Ping Luo (The First Affiliated Hospital of Jinan University, Guangzhou, China)

© 2025 Zhu H et al. *JAMA Internal Medicine*.

3. Zhan-Yu Cai (The First Affiliated Hospital of Jinan University, Guangzhou, China)
4. Ai-Dong Zhang (The First Affiliated Hospital of Jinan University, Guangzhou, China)
5. Sheng-Ming Liu (The First Affiliated Hospital of Jinan University, Guangzhou, China)
6. Yi Zhou (The First Affiliated Hospital of Jinan University, Guangzhou, China)
7. Xiao-Min Xiao (The First Affiliated Hospital of Jinan University, Guangzhou, China)
8. Yue-Ping Liu (The First Affiliated Hospital of Jinan University, Guangzhou, China)
9. Hai-Ping Jiang (The First Affiliated Hospital of Jinan University, Guangzhou, China)
10. Wei-Ju Chen (The First Affiliated Hospital of Jinan University, Guangzhou, China)
11. Bo-Qiao Li (The First Affiliated Hospital of Jinan University, Guangzhou, China)
12. Xiao-Song Wu (The First Affiliated Hospital of Jinan University, Guangzhou, China)
13. Dong-Xia Wu (The First Affiliated Hospital of Jinan University, Guangzhou, China)
14. Shao-Hua Chen (The First Affiliated Hospital of Jinan University, Guangzhou, China)
15. Yi-Ping Qiu (The First Affiliated Hospital of Jinan University, Guangzhou, China)
16. Guo-Hua Chen (The First Affiliated Hospital of Jinan University, Guangzhou, China)
17. Juan Wang (The First Affiliated Hospital of Jinan University, Guangzhou, China)
18. Shen Zheng (The First Affiliated Hospital of Jinan University, Guangzhou, China)

© 2025 Zhu H et al. *JAMA Internal Medicine*.

eMethods

1 Overview of Study Procedures:

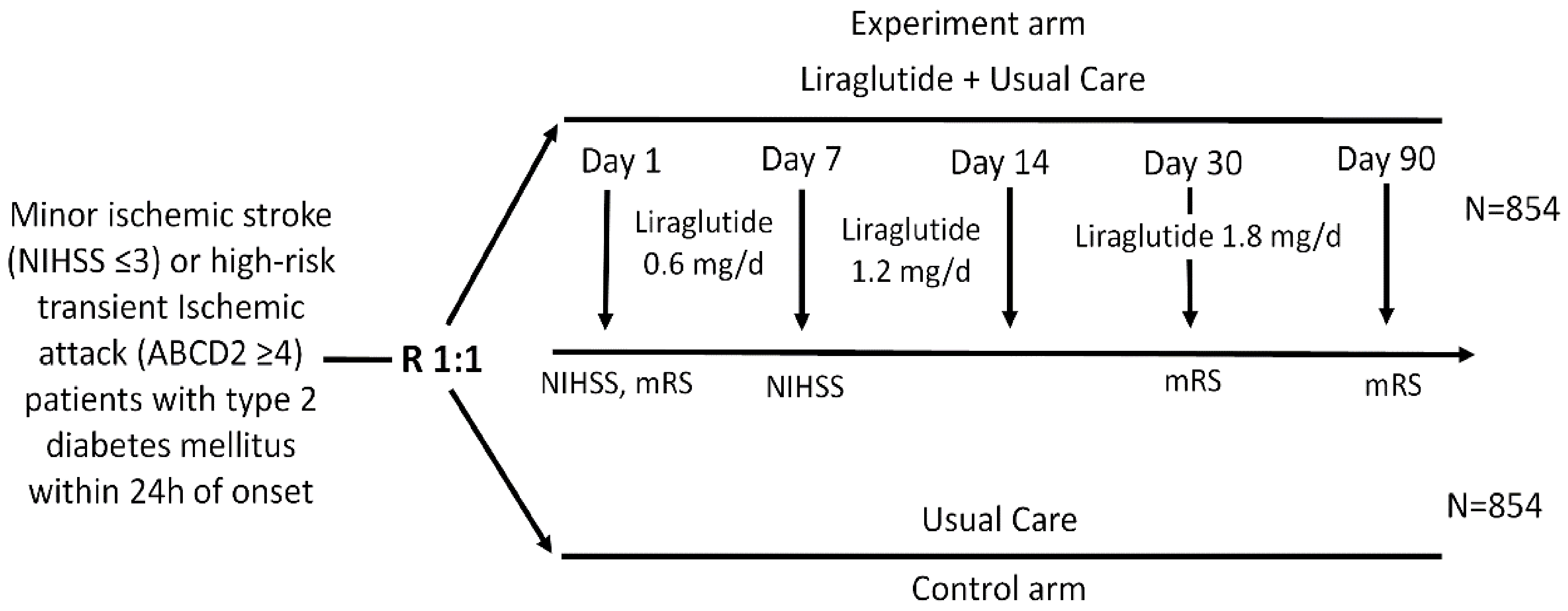

Abbreviation: mRS = modified Rankin Scale; NIHSS = National Institutes of Health Stroke Scale.

© 2025 Zhu H et al. *JAMA Internal Medicine*.

2 Inclusion and Exclusion Criteria:

Inclusion criteria:

1. Adult patients (male or female, ≥50 years).
2. Acute ischemic stroke (National Institutes of Health Stroke Scale [NIHSS] ≤3 at the time of randomization) or high-risk transient ischemic attack (ABCD2 ≥4 at the time of randomization) patients with type 2 diabetes mellitus within 24 hours of symptom onset.
3. First stroke or prior stroke without sequel (modified Rankin Scale ≤1) and does not affect the NIHSS score.
4. Informed consent signed.

Exclusion criteria:

1. Diagnosis of intracranial hemorrhagic diseases on baseline computed tomography.
2. Iatrogenic or cardiogenic stroke.
3. Patients undergoing thrombolysis or endovascular treatment.
4. Regular glucagon-like peptide-1 RA use in the last 90 days.
5. Family/personal history of multiple endocrine neoplasia type 2 or familial medullary thyroid carcinoma.
6. Patients with pancreatitis or previous history of pancreatitis, inflammatory bowel disease, or gastroparesis.
7. Pregnant, lactating women, or patients likely or planning to become pregnant.
8.Allergic to liraglutide or excipients.
9. Congestive heart failure (New York Heart Association class III-IV).
10. Severe liver or kidney dysfunction (aspartate transaminase/alanine transaminase ratio and serum creatinine are 3 times higher than the normal upper limit).
11. Participated in other clinical trials of drugs within 3 months.
12. Researchers deem patients unsuitable for this study.

© 2025 Zhu H et al. *JAMA Internal Medicine*.

3 Outcome assessment

Structure interview for telephone assessment: a structured telephone interview and interview algorithm was used as reported in a previous study[1].

The primary outcome assessment: the primary outcome was adjudicated by an outcome committee who blinded to the group assignments. In the event of disagreement, an outcome committee decided by vote.

The mRS assessment: the blinded mRS assessors were trained uniformly on how to evaluate the mRS based on a structured telephone interview algorithm. After the training, 20 examples based on the neurological function description of patients were used to assess this score and assessors were certified when the intraclass correlation coefficient was ≥ 0.95. The mRS Score had to be performed at a 90-day visit by an mRS-certified assessor who was unaware of the treatment assignments.

Reference

1. Wilson JT, Hareendran A, Grant M, et al. Improving the assessment of outcomes in stroke: use of a structured interview to assign grades on the modified Rankin Scale. *Stroke*. Sep 2002;33(9):2243-6. doi:10.1161/01.str.0000027437.22450.bd

© 2025 Zhu H et al. *JAMA Internal Medicine*.

4. Clinicaltrials.gov registration

LAMP was a multicenter, controlled, prospective, randomized, open-label, blinded endpoint (PROBE) trial registered at clinicaltrials.gov on May 10, 2019 (NCT03948347). The trial was initially set up on June 10, 2019, and their first patient was recruited on June 25, 2019. The last patient was recruited on December 27, 2023, and finished on March 24, 2024. The recruitment status was changed to completed on December 31, 2023, and the study status was changed to completed on March 24, 2024.

© 2025 Zhu H et al. *JAMA Internal Medicine*.

eFigure 1 Trial Profile

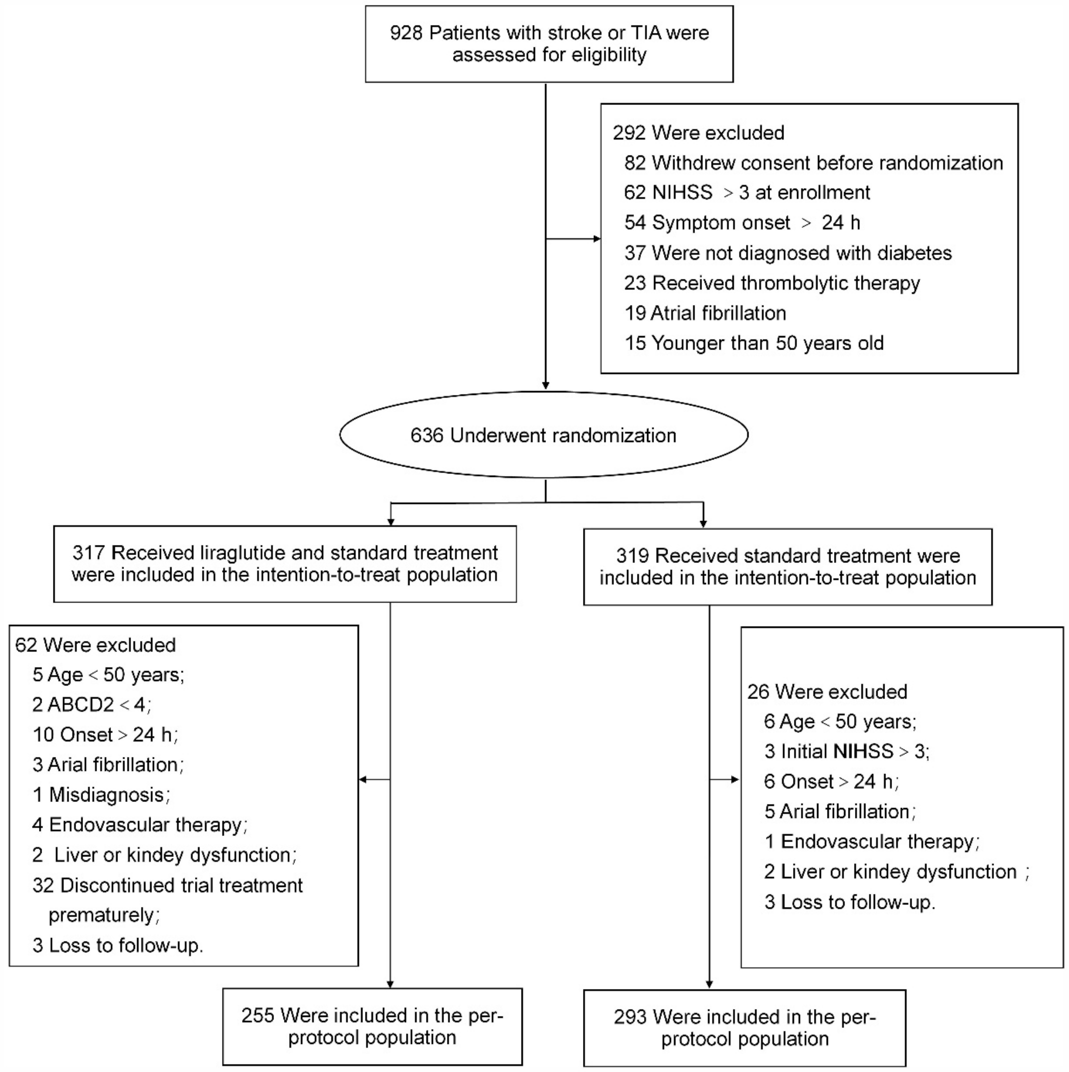

This figure shows the overall patient flow in the trial, including the full analysis set population, and the perprotocol population.

© 2025 Zhu H et al. *JAMA Internal Medicine*.

eFigure 2 Distribution of Modified Rankin Scale Scores at 90 Days in the Intention-to-Treat

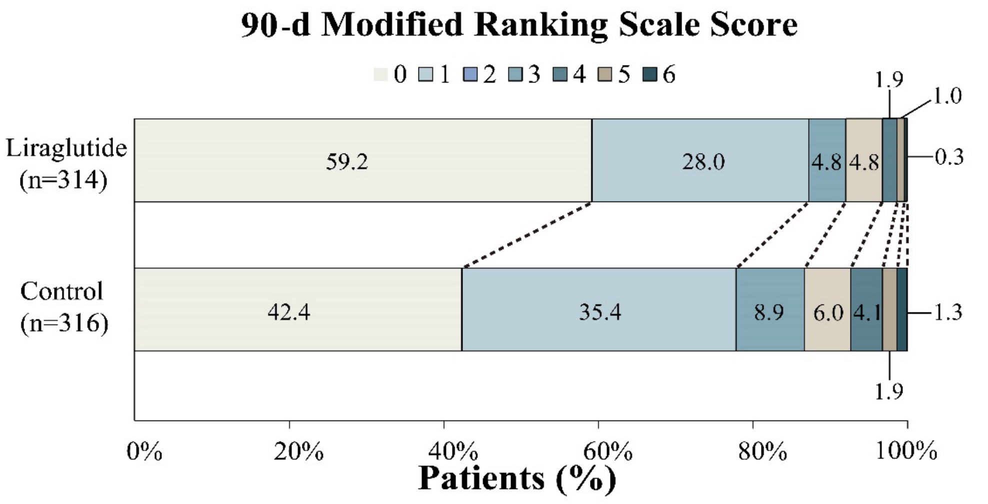

The raw distribution of scores is shown. Scores ranged from 0 to 6, where 0 = no symptoms, 1 = symptoms without clinically significant disability, 2 = slight disability, 3 = moderate disability, 4 = moderately severe disability, 5 = severe disability, and 6 = death.

eFigure 3 Distribution of Modified Rankin Scale Scores at 90 Days in the Per-Protocol Analysis

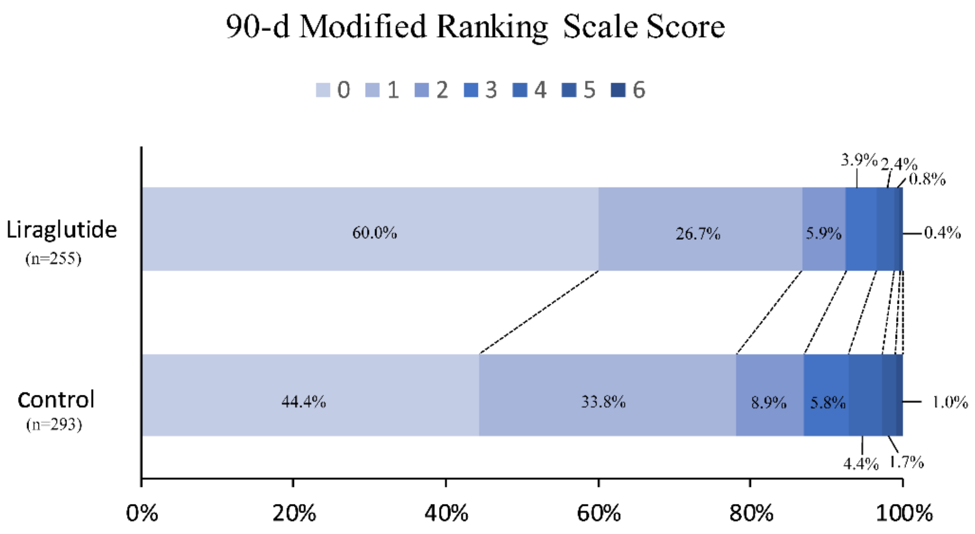

The raw distribution of scores is shown. Scores ranged from 0 to 6. 0 = no symptoms, 1 = symptoms without clinically significant disability, 2 = slight disability, 3 = moderate disability, 4 = moderately severe disability, 5 = severe disability, and 6 = death.

© 2025 Zhu H et al. *JAMA Internal Medicine*.

eFigure 4 Primary Outcome by Prespecified Subgroups in the Per-Protocol Analysis

Variable	Count	Percent(%)	HR (95% CI)	P.value	P.for.interaction
Age					0.222
<65	291	53.1	0.38 (0.16 to 0.9)	0.029	
>=65	257	46.9	0.74 (0.38 to 1.42)	0.366	
Sex					0.234
Male	351	64.1	0.42 (0.21 to 0.83)	0.013	
Female	197	35.9	0.8 (0.35 to 1.83)	0.604	
Body mass index					0.173
<25	339	63.2	0.38 (0.18 to 0.81)	0.013	
>=25	197	36.8	0.82 (0.37 to 1.81)	0.626	
Qualifying event					1
stroke	531	96.9	0.55 (0.33 to 0.93)	0.026	
TIA	17	3.1			
Previous hypertension					0.457
Yes	382	69.7	0.48 (0.25 to 0.89)	0.021	
No	166	30.3	0.74 (0.29 to 1.92)	0.539	
Previous ischemic stroke					0.615
Yes	149	27.2	0.66 (0.26 to 1.66)	0.378	
No	399	72.8	0.5 (0.27 to 0.95)	0.033	
Previous TIA					0.63
Yes	18	3.3	0.88 (0.12 to 6.27)	0.9	
No	530	96.7	0.52 (0.3 to 0.9)	0.019	
Current smoker					0.03
Yes	180	32.8	0.18 (0.05 to 0.61)	0.006	
No	368	67.2	0.81 (0.44 to 1.49)	0.503	
Overall	548	100	0.54 (0.32 to 0.92)	0.022	

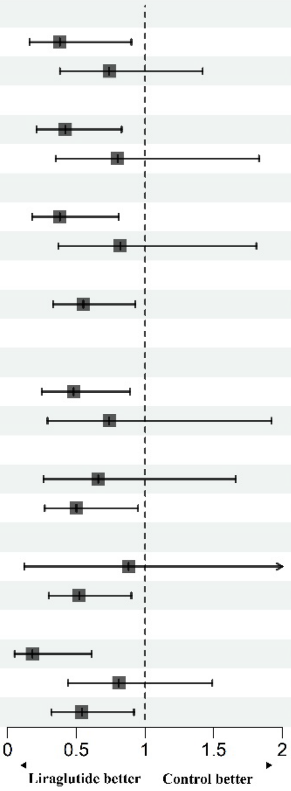

A Cox proportional hazards model was fitted to each subgroup separately by modeling the primary outcome with treatment, subgroup, and treatment-by-subgroup interaction terms. The primary outcome is the percentage of patients developing a new stroke (ischemic or hemorrhagic stroke) within 90 days. For subcategories, black squares represent point estimates (with the area of the square proportional to the number of events) and horizontal lines represent the 95% CI.

© 2025 Zhu H et al. *JAMA Internal Medicine*.

eTable 1 Criteria for the diagnosis of diabetes

FPG ≥126 mg/dL (7.0 mmol/L). Fasting is defined as no caloric intake for at least 8 h.*

OR

2-h PG ⩾200 mg/dL (11.1 mmol/L) during OGTT. The test should be performed as described by the WHO, using a glucose load containing the equivalent of 75-g anhydrous glucose dissolved in water. *

OR

A1C ⩾6.5% (48 mmol/mol). The test should be performed in a laboratory using a method that is NGSP certified and standardized to the DCCT assay. *

OR

In a patient with classic symptoms of hyperglycemia or hyperglycemic crisis, a random plasma glucose ⩾200 mg/dL (11.1 mmol/L).

*In the absence of unequivocal hyperglycemia, diagnosis requires two abnormal test results from the same sample or in two separate test samples.

© 2025 Zhu H et al. *JAMA Internal Medicine*.

eTable 2 Definitions of stroke events and vascular events

Stroke	Acute symptoms and signs of neurological defect caused by sudden abnormality of the blood supply. Damage of focal or whole brain, spinal or retinal vascular damage, which is related to cerebral circulation disorder.
Ischemic stroke	Definitions: (1) Symptoms or imaging evidence of acute newly onset focal neurologic deficit last for more than 24 hours after excluding other non-ischemic reasons, such as brain infection, head trauma, brain tumor, epilepsy, severe metabolic diseases, degeneration diseases or adverse effect of medications; or (2) Acute brain or retinal ischemic event with focal symptoms or signs lasts for less than 24 hours after excluding other causes with imaging evidence of new infarction; or (3) Progression of original vascular ischemic stroke (NIHSS increased≥4 from baseline score after excluding hemorrhagic transformation or symptomatic intracerebral hemorrhage after cerebral infarction) lasts over 24 hours with new ischemic lesion on brain MRI or CT. Which would be classified by ASCO etiology standard.
Transient ischemic attack	A brief episode of neurological dysfunction caused by focal brain or retinal ischemia, with clinical symptoms typically lasting less than 24 hours, and without evidence of acute infarction, after excluding other non-ischemic reasons, such as brain infection, head trauma, brain tumor, epilepsy, severe metabolic diseases, degeneration diseases or adverse effect of medications.
Hemorrhagic stroke	Hemorrhagic stroke was defined as focal or whole brain or spine damage caused by non-traumatic bleeding into the brain parenchyma, intraventricular or subarachnoid.
Myocardial infarction	Third universal definition of myocardial infarction（Thygesen 2012） The term acute myocardial infarction (MI) should be used when there is evidence of myocardial necrosis in a clinical setting consistent with acute myocardial ischemia. Under these conditions

© 2025 Zhu H et al. *JAMA Internal Medicine*.

	any one of the following criteria meets the diagnosis for MI: 1、Detection of a rise and/or fall of cardiac biomarker values [preferably cardiac troponin (cTn)] with at least one value above the 99th percentile upper reference limit (URL) and with at least one of the following: (1) Symptoms of ischemia. (2) New or presumed new significant ST-segment–T wave (ST–T) changes or new left bundle branch block (LBBB). (3) Development of pathological Q waves in the ECG. (4) Imaging evidence of new loss of viable myocardium or new regional wall motion abnormality (5) Identification of an intracoronary thrombus by angiography or autopsy. 2、Cardiac death with symptoms suggestive of myocardial ischemia and presumed new ischemic ECG changes or new LBBB, but death occurred before cardiac biomarkers were obtained, or before cardiac biomarker values would be increased. 3、Percutaneous coronary intervention (PCI) related MI is arbitrarily defined by elevation of cTn values (>5×99th percentile URL) in patients with normal baseline values (≤99th percentile URL) or a rise of cTn values >20% if the baseline values are elevated and are stable or falling. In addition, either (1) symptoms suggestive of myocardial ischemia or (2) new ischemic ECG changes or (3) angiographic findings consistent with a procedural complication or (4) imaging demonstration of new loss of viable myocardium or new regional wall motion abnormality are required. 4、Stent thrombosis associated with MI when detected by coronary angiography or autopsy in the setting of myocardial ischemia and with a rise and/or fall of cardiac biomarker values with at least one value above the 99th percentile URL. 5、Coronary artery bypass grafting (CABG) related MI is arbitrarily defined by elevation of cardiac biomarker values (>10×99th percentile URL) in patients with normal baseline cTn values (≤99th percentile URL). In addition, either (1)

© 2025 Zhu H et al. *JAMA Internal Medicine*.

	new pathological Q waves or new LBBB, or (2) angiographic documented new graft or new native coronary artery occlusion, or (3) imaging evidence of new loss of viable myocardium or new regional wall motion abnormality.
Vascular death	Vascular death include death due to stroke, cardiac sudden death, death caused by acute myocardial infarction, death caused by heart failure, death caused by pulmonary embolism, death caused by cardial/cerebral interventions or operations (not caused by myocardial infarction) and death caused by other cardiovascular Vascular death diseases. (Arrhythmia irrelevant to cardiac sudden death, rupture of aortic aneurysm or peripheral artery disease). Unexplained death happened within 30 days after stroke, myocardial infarction or cardiovascular/cerebral vascular operation will be considered as stroke, myocardial infarction and accidental death caused by operation separately.

© 2025 Zhu H et al. *JAMA Internal Medicine*.

eTable 3 Baseline Characteristics of the Population in the Per-Protocol Analysis

eTable 3. Patient Baseline Characteristics in the PPS[a]

Characteristic	Control (N=293)	Liraglutide (N=255)
**Median age ([IQR]) — yr**	65.0 [59.0, 71.0]	63.00 [57.0, 69.5]
**Female sex — no.(%)**	101 (34.5)	96（37.6）
**Median BMI [b] ([IQR])**	23.6 [22.0, 26.0]	24.2 [22.4, 26.0]
**Median HbA1c level on admission ([IQR]) — %**	8.2 [6.8, 9.9]	8.0 [7.0, 9.8]
**Median random blood glucose on admission ([IQR]) — mg/dL**	205.4 [147.8, 288.3]	217.8 [147.8, 272.1]
**Medical history — no. (%)**		
**Diabetes mellitus**	263 (89.8)	232 (91.0)
**Hypertension**	204 (69.6)	178 (69.8)
**Dyslipidemia**	36 (12.3)	39 (15.3)
**Previous ischemic stroke**	83 (28.3)	66 (25.9)
**Previous TIA**	9 (3.1)	9 (3.5)
**Coronary heart disease**	25 (8.5)	22 (8.6)
**Median blood pressure — mm Hg**		
**Systolic**	154.0 [140.0, 166.0]	152.00 [139.0, 166.0]
**Diastolic**	87.4±13.2	89.0±12.2
**Current smoker — no. (%)**	97 (33.1)	83 (32.5)
**Qualifying event — no. (%)**		
**Stroke**	286 (97.6)	246 (96.5)
**TIA**	7 (2.4)	9(3.5)
**TOAST classification [C] — no. (%)**		
**Large-artery atherosclerosis**	145 (50.7)	118 (48.0)
**Cardioembolic**	0 (0.0)	0 (0.0)
**Small vessel occlusion**	132 (46.2)	121 (49.2)
**Stroke of other determined etiology**	0 (0.0)	0 (0.0)
**Stroke of undetermined etiology**	9 (3.1)	7 (2.8)
**Median NIHSS score in patients with qualifying ischemic stroke[d] ([IQR])**	2.0 [1.0, 3.0]	2.00 [1.0, 2.0]
**Median ABCD2 score in patients with qualifying TIA[e] ([IQR])**	4.0 [4.0, 4.8]	5.0 [4.3, 5.0]
**Antithrombotic therapy— no. (%)**		
**Dual antiplatelet therapy**	220(76.9)	196(78.4)
**Single antiplatelet therapy**	66(23.1)	54(21.6)
**Lipid-lowering therapy— no. (%)**		
**Yes**	269 (91.8)	237 (92.9)
**No**	24 (8.2）	18 (7.1)

© 2025 Zhu H et al. *JAMA Internal Medicine*.

**Antihypertensive therapy— no. (%)**		
**Yes**	165(56.3)	147(57.6)
**No**	128(43.7)	108(42.4)

Data are n (%), median (IQR), or mean ± standard. IQR = interquartile range; BMI = body mass index; TOAST = Trial of ORG 10172 in Acute Stroke Treatment; TIA = transient ischemic attack; NIHSS = National Institutes of Health Stroke Scale.

SI conversions: To convert random blood glucose and FBG to millimoles per liter, multiply by 0.0555.

[a] Only patients who fully met the inclusion and exclusion criteria were included.

[b] BMI is the weight in kilograms divided by the square of the height in meters.

[c] The presumed stroke cause was classified according to the Trial of ORG 10172 in Acute Stroke Treatment (TOAST) criteria, using clinical findings, brain imaging, and laboratory tests. A total of 16 patients diagnosed with TIA lacked TOAST classification information

[d] NIHSS is a standardized neurologic examination comprising 15 questions covering 11 specific functions scored on a scale of 0 to 4, where 0 indicates normal functioning and 4 indicates complete impairment; a score of 42 indicates death.

[e] Among patients with TIA, the qualifying score was 4 or more on the ABCD2 scale, which ranges from 0 to 7, with higher scores indicating a greater risk of stroke. The scale is used to estimate the risk of recurrent stroke after a TIA based on age, blood pressure, clinical features, duration of symptoms, and presence of diabetes.

© 2025 Zhu H et al. *JAMA Internal Medicine*.

eTable 4 The patient's glycemic control and antidiabetic drug use status in Full Analysis Set

Characteristic	Control (N=319)	Liraglutide (N=317)
Glycemic control		
Median FBG on the seventh day ([IQR]) —mg/dL	124.3 (99.8-160.2)	121.3 (99.1-149.0)
Median PBG on the seventh day ([IQR]) —mg/dL	195.9 (171.0-230.6)	185.8 (152.4-219.1)
Median post-discharge FBG ([IQR]) —mg/dL	132.3 (119.8-149.2)	127.9 (116.8-140.5)
Median post-discharge PBG levels ([IQR]) —mg/dL	171.7 (153.2-204.5)	167.2 (146.3-193.7)
Antidiabetic therapy— no. (%)	308(96.6)	306(96.5)
Metformin	174(54.5)	127(40.1)
Acarbose	147(46.1)	88(27.8)
Insulin	111(34.8)	51(16.1)
Sulfonylureas	73(22.9)	26(8.2)
Other [a]	23(7.2)	15(4.7)

Abbreviations: FAS, full analysis set IQR, interquartile range FBG, Fasting blood glucose PBG, Postprandial blood glucose

SI conversions: To convert FBG and PBG to millimoles per liter, multiply by 0.0555.

[a] non-sulfonylurea insulin secretagogues and thiazolidinediones.

© 2025 Zhu H et al. *JAMA Internal Medicine.*

eTable 5 Trial Outcomes in the Per-Protocol Analysis.

eTable 5. Efficacy and Safety Outcomes.

Outcome	Control (n=293)		Liraglutide (n=255)		Hazard Ratio [a] or Odds Ratio [b] (95% CI) [c]	P Value
	Patients with Event no.	Incidence %	Patients with Event no.	Incidence %		
**Primary outcome**						
Stroke	43	14.7	21	8.2	0.54(0.32-0.92)	0.02
**Secondary outcomes**						
Vascular event [d] within 90 days	49	16.7	22	8.6	0.50(0.30-0.82)	0.01
mRS [e]						
mRS≤1	229	78.2	221	86.7	1.82(1.16-2.89)	0.01
mRS≤2	255	87.0	236	92.5	1.85(1.05-3.36)	0.04
**Safety outcomes**						
sICH [f]	2	0.7	1	0.4	0.54(0.05-6.00)	0.60
Gastrointestinal disorders	12	4.1	50	19.6	5.02(2.67-9.42)	< 0.001
Hypoglycemia [j]	24	8.2	17	6.7	0.77(0.42-1.44)	0.40
Pneumonia	2	0.7	2	0.8	1.11(0.16-7.86)	0.90
Acute pancreatitis	0	0	0	0	NA	NA
Death	3	1.0	1	0.4	0.36(0.04-3.43)	0.30

CI = confidence interval; mRS = modified Rankin Scale; sICH = symptomatic intracerebral hemorrhage.

[a] Calculated using the Cox regression model.

[b] Calculated using a generalized linear model

[c] The common odds ratio is shown for mRS. Hazard ratios are shown for other outcomes.

[d] Vascular events included ischemic stroke, hemorrhagic stroke, TIA, myocardial infarction, and vascular death

[e] mRS scores range from 0 to 6: 0, no symptoms, 1 = symptoms without clinically significant disability, 2 = slight disability, 3 = moderate disability, 4 = moderately severe disability, 5 = severe disability; and 6 = death.

[f] sICH based on the ECASS II study definition

[j] Hypoglycemic events were defined as blood glucose levels below 3.9mmo/L

© 2025 Zhu H et al. *JAMA Internal Medicine*.
