# Supplementary material for: Liraglutide in Acute Minor Ischemic Stroke or High-Risk Transient Ischemic Attack With Type 2 Diabetes: The LAMP Randomized Clinical Trial
Source: JAMA Intern Med. 2025 Nov 3;186(1):46–54. doi: 10.1001/jamainternmed.2025.5684 (PMC12584062; doi:10.1001/jamainternmed.2025.5684)
Supplement: Supplement 4. — Nonauthors collaborators [file jamainternmed-e255684-s004.pdf]

Supplemental Online Content: Nonauthor Collaborators
*First name, last name, and suffix (if applicable) are required and will appear in PubMed.

*Group Name(s): The LAMP Investigators							
***First Name and Middle Initial(s)**	***Last Name**	***Suffix (eg, Jr, III)**	Academic Degrees	Institution	Location (city, state/province, country)	Role or Contribution, eg, chair, principal investigator	Group (if more than 1 Group listed in the byline) and/or Subgroup (eg, Steering Committee)
Wen-Yan	Zhuo		MD	Department of Neurology, Zhuhai People's Hospital	Zhuhai, Guangdong, China	principal investigator	Steering Committee
Qing-Yu	Shen		MD	Department of Neurology, Sun Yat-sen Memorial Hospital, Sun Yat-sen University	Guangzhou, Guangdong, China	principal investigator	Steering Committee
Jian	Xia		MD	Department of Neurology, Xiangya Hospital, Central South University	Changsha, Hunan, China	principal investigator	Steering Committee
Zhen-Gang	Cha		MD	The First Affiliated Hospital of Jinan University	Guangzhou, China	Chairman	Institutional Human Research Ethics Committee
Liang-Ping	Luo		MD	The First Affiliated Hospital of Jinan University	Guangzhou, China	Member	Institutional Human Research Ethics Committee
Zhan-Yu	Cai		MD	The First Affiliated Hospital of Jinan University	Guangzhou, China	Member	Institutional Human Research Ethics Committee
Ai-Dong	Zhang		MD	The First Affiliated Hospital of Jinan University	Guangzhou, China	Member	Institutional Human Research Ethics Committee
Sheng-Ming	Liu		MD	The First Affiliated Hospital of Jinan University	Guangzhou, China	Member	Institutional Human Research Ethics Committee
Yi	Zhou		MD	The First Affiliated Hospital of Jinan University	Guangzhou, China	Member	Institutional Human Research Ethics Committee
Xiao-Min	Xiao		MD	The First Affiliated Hospital of Jinan University	Guangzhou, China	Member	Institutional Human Research Ethics Committee

Supplemental Online Content: Nonauthor Collaborators
*First name, last name, and suffix (if applicable) are required and will appear in PubMed.

***First Name and Middle Initial(s)**	***Last Name**	***Suffix (eg, Jr, III)**	Academic Degrees	Institution	Location (city, state/province, country)	Role or Contribution, eg, chair, principal investigator	Group (if more than 1 Group listed in the byline) and/or Subgroup (eg, Steering Committee)
Yue-Ping	Liu		MD	The First Affiliated Hospital of Jinan University	Guangzhou, China	Member	Institutional Human Research Ethics Committee
Hai-Ping	Jiang		MD	The First Affiliated Hospital of Jinan University	Guangzhou, China	Member	Institutional Human Research Ethics Committee
Wei-Ju	Chen		MD	The First Affiliated Hospital of Jinan University	Guangzhou, China	Member	Institutional Human Research Ethics Committee
Bo-Qiao	Li		MD	The First Affiliated Hospital of Jinan University	Guangzhou, China	Member	Institutional Human Research Ethics Committee
Xiao-Song	Wu		MD	The First Affiliated Hospital of Jinan University	Guangzhou, China	Member	Institutional Human Research Ethics Committee
Dong-Xia	Wu		MD	The First Affiliated Hospital of Jinan University	Guangzhou, China	Member	Institutional Human Research Ethics Committee
Shao-Hua	Chen		MD	The First Affiliated Hospital of Jinan University	Guangzhou, China	Member	Institutional Human Research Ethics Committee
Yi-Ping	Qiu		MD	The First Affiliated Hospital of Jinan University	Guangzhou, China	Member	Institutional Human Research Ethics Committee
Guo-Hua	Chen		MD	The First Affiliated Hospital of Jinan University	Guangzhou, China	Member	Institutional Human Research Ethics Committee
Juan	Wang		MD	The First Affiliated Hospital of Jinan University	Guangzhou, China	Member	Institutional Human Research Ethics Committee

Supplemental Online Content: Nonauthor Collaborators
*First name, last name, and suffix (if applicable) are required and will appear in PubMed.

***First Name and Middle Initial(s)**	***Last Name**	***Suffix (eg, Jr, III)**	Academic Degrees	Institution	Location (city, state/province, country)	Role or Contribution, eg, chair, principal investigator	Group (if more than 1 Group listed in the byline) and/or Subgroup (eg, Steering Committee)
Shen	Zheng		MD	The First Affiliated Hospital of Jinan University	Guangzhou, China	Member	Institutional Human Research Ethics Committee
David	Wang		MD	St. Joseph's Hospital and Medical Center	Phoenix, USA	Chair	Data Monitoring Committee
An-Xin	Wang		PhD	Tiantan Hospital, Capital Medical University	Beijing, China	Medical Statistics	Data Monitoring Committee
Jun	Lv		PhD	The First Affiliated Hospital of Jinan University	Guangzhou, Guangdong, China	Medical Statistics	Data Monitoring Committee
Yan-Sheng	Li		MD	Department of Neurology, Renji Hospital affiliated to Shanghai Jiaotong University School of Medicine	Shanghai, China	Chair	Clinical Events Committee
Kang-Ning	Chen		MD	Department of Neurology, The First Affiliated Hospital of Army Medical University	Chongqing, China	Member	Clinical Events Committee
Bo	Hu		MD	Union Hospital Affiliated to Tongji Medical College, Huazhong University of Science and Technology	Wuhan, Hubei, China	Member	Clinical Events Committee
Yong-Shun	Zhang		MD	Department of Neurology, the Sixth Affiliated Hospital of Jinan University	Dongguan, Guangdong, China	principal investigator	
Qiao-Wei	Li		MD	Department of Neurology, Panyu Central Hospital, Guangzhou	Guangzhou, Guangdong, China	principal investigator	
Wei-Jian	Xie		MD	Department of Neurology, He Xian Memorial Hospital, Panyu District, Guangzhou	Guangzhou, Guangdong, China	principal investigator	

Supplemental Online Content: Nonauthor Collaborators
*First name, last name, and suffix (if applicable) are required and will appear in PubMed.

***First Name and Middle Initial(s)**	***Last Name**	***Suffix (eg, Jr, III)**	Academic Degrees	Institution	Location (city, state/province, country)	Role or Contribution, eg, chair, principal investigator	Group (if more than 1 Group listed in the byline) and/or Subgroup (eg, Steering Committee)
Ji	Liang		MD	Department of Neurology, Changde Hospital, Xiangya School of Medicine, Central South University (The first people's hospital of Changde city)	Changde, Hunan, China	principal investigator	
Shen-Wen	He		MD	Department of Neurology, Shunde Hospital Affiliated to Jinan University	Shunde, Guangdong, China	principal investigator	
