# Supplementary material for: Liraglutide in Acute Minor Ischemic Stroke or High-Risk Transient Ischemic Attack With Type 2 Diabetes: The LAMP Randomized Clinical Trial
Source: JAMA Intern Med. 2025 Nov 3;186(1):46–54. doi: 10.1001/jamainternmed.2025.5684 (PMC12584062; doi:10.1001/jamainternmed.2025.5684)
Supplement: Supplement 5. — Data sharing statement [file jamainternmed-e255684-s005.pdf]

Data Sharing Statement

Zhu. Liraglutide in Acute Minor Ischemic Stroke or High-Risk Transient Ischemic Attack With Type 2 Diabetes. *JAMA Intern Med*. Published November 03, 2025.
doi:10.1001/jamainternmed.2025.5684

Data
Additional Information: Liraglutide in Acute Minor Ischemic Stroke or High-risk Transient Ischemic Attack Patients With Type 2 Diabetes Mellitus (LAMP)
URL：https://clinicaltrials.gov/study/NCT03948347?term=NCT03948347&rank=1
ClinicalTrials.gov Identifier: NCT03948347
Data available: No
